# Salivary microbiome, socioeconomic status, and cognitive performance: Triangulating the relationship

**DOI:** 10.1371/journal.pone.0361602

**Published:** 2026-11-02

**Authors:** Oxana Y. Naumova, Pavel V. Dobrynin, Griffin Litwin, Sergey Y. Rychkov, Michael P. Milham, Elena L. Grigorenko

**Affiliations:** 1 Department of Psychology, University of Houston, Houston, Texas, United States of America; 2 Vavilov Institute of General Genetics, Russian Academy of Sciences RAS, Moscow, Russia; 3 Hewlett Packard Enterprise Data Science Institute, University of Houston, Houston, Texas, United States of America; 4 Center for the Developing Brain, Child Mind Institute, New York, United States of America; 5 Center for Biomedical Imaging and Neuromodulation, Nathan Kline Institute, Orangeburg, New York, United States of America; 6 Departments of Molecular and Human Genetics and Pediatrics, Baylor College of Medicine, Houston, Texas, United States of America; 7 Child Study Center, Yale University, New Haven, Connecticut, United States of America; University of Granada: Universidad de Granada, SPAIN

## Abstract

Research increasingly demonstrates that endogenous bacterial communities (microbiomes) are associated with both cognitive functioning and socioeconomic status (SES). In this study, we used a metagenomics approach to characterize oral microbiome diversity in Hispanic and African American children aged 6–16 years. We examined how microbiome composition and SES were related to cognitive performance, as assessed by the Wechsler Intelligence Scale for Children (WISC). Our findings indicate that SES and the microbiome contributed complementary, comparably sized influences on cognitive functioning. Together, variability in conventional SES indicators and microbiome composition accounted for approximately 10% of the variability in WISC scores. These results highlight the importance of integrating both social and biological determinants of health when studying cognitive performance and other psychological outcomes.

## Introduction

A substantial body of research has examined the relationship between socioeconomic status (SES), typically indexed by parental education, income, and occupation, and cognitive functioning, assessed by standardized psychological tests. Consistently, children and adolescents from higher-SES backgrounds outperform their lower-SES peers on tests of intelligence and academic achievement [1]. These disparities are substantiated by neurobiological evidence: SES is associated with structural and functional differences in cognitive and affective neural systems that shape outcomes in cognition, academic performance, and mental health. Such SES—brain associations arise through multiple pathways, including prenatal factors, early caregiving environments, and a range of distal and proximal child factors [2]. Collectively or individually, these factors can generate chronic stress that alters brain development and function [2,3]. SES also influences access to cognitively enriching environments, better educational opportunities and extracurricular activities, healthier nutrition and more advanced healthcare, lifestyle habits, and cognitive engagement, all of which contribute to differences that emerge in childhood and persist across the lifespan [4].

SES encompasses a broad spectrum of factors that impact all aspects of human physical and mental health, including the human microbiota—the collective of microorganisms that live in association with various body parts and organs. Microbiota composition reflects stable dietary patterns, lifestyle, and health behaviors (e.g., nutrition, sanitation, stress, healthcare access); all of which vary systematically by SES. Consequently, SES shapes the diversity and structure of the microbiome (i.e., the microbiota and their genes, as well as the interactions between them and their environment) [5–7]. Given its omnipresence, microbiota is embedded in all organismal functions and, therefore, may be associated with characteristics of development in general and neuro- and cognitive development in particular.

Over the past decade, the interaction between the gut microbiota, typically ascertained through stool specimens, and the central nervous system has been studied through the “microbiota-gut-brain axis,” MGBA [8]. MGBA is underpinned by the interplay between the nervous, endocrine, and immune systems, and it impacts processes such as neuronal modulation, immune response, and hormone release [9–11]. MGBA has been implicated in various neurodevelopmental and neurodegenerative disorders [12–14].

In addition to the gut, another large microbial community associated with the digestive tract is the oral microbiota, which can be ascertained through salivary specimens [5]. Recent studies have demonstrated the existence of the oral–gut microbiome axis, highlighting the oral-to-gut microbial transmission of taxa active along the gastrointestinal tract that can influence intestinal immunity and regulate pathogenesis [15–17]. Although the gut microbiome has received the most attention, the oral microbiome is increasingly recognized as relevant to systemic health conditions, including cardiovascular disease, bacterial pneumonia, diabetes, and atherosclerosis, among others [15,18,19]. Alterations in the oral microbiome have been associated with neuropsychiatric disorders broadly [20], as well as with schizophrenia onset [21] and cognitive decline associated with dementia and Alzheimer’s disease [22–24].

Growing evidence suggests that specific features of microbial communities—whether in the oral or gut microbiome—are associated with cognitive functioning. However, most findings come from case-control studies of clinical populations, such as individuals with Alzheimer’s disease, whereas research examining microbiota-cognition associations in normative samples remains limited. To date, only two community-based studies have associated gut microbiome composition with cognitive performance in middle-aged adults [25] and school-age children [26]. For the oral microbiome, just one population-based study has examined its relationship with cognition (executive function and memory) in a nationally representative sample of older US adults [27]. Our study, therefore, addresses a significant gap by investigating associations between the oral microbiota and cognitive functioning in youth.

In summary, both SES and microbiome have been associated with cognition through distinct yet interconnected pathways involving brain development, stress physiology, and mental health. However, microbiome-cognition studies typically treat SES as a confounding variable rather than a parallel explanatory factor. In this study, we conceptualize SES and microbiome as semi-independent factors, reflecting external and internal environments, respectively, that jointly shape cognitive functioning. Our central hypothesis is that SES and microbiome exert complementary influences of similar magnitude on cognitive performance. To our knowledge, this work represents one of the first attempts to integrate conventional SES indicators and oral-microbiome profiles within a unified framework for understanding predictors of cognitive functioning.

## Results

### Oral microbiota composition and variability

A total of 237 genera were identified in the saliva specimens. Of those, 23 genera were detected across all 200 (Table 1) and 83—in at least 50% of individuals (S1 Table in S1 File). According to published data [28], *Streptococcus, Neisseria, Prevotella, Veillonella, Haemophilus, Rothia,* and *Ca. Saccharibacteria* genera were the core members of the salivary microbiome. There was no significant difference in either α-diversity or distinct taxa abundance across sex and ethnic groups. However, among the 83 most common taxa, 7 and 18 showed a nominally significant association with sex and ethnicity, respectively (S2 Table in S1 File). No significant association was found between Simpson’s D and age, while Richness was positively associated with age: *β* = .2958; SD = .0679; 95% CI = .1619−.4296; t(3;196) = 4.29; *p* < .001. Genera-specific analysis based on Spearman’s correlation (R_s_) revealed nominally significant associations between children’s age and the relative abundances of 24 genera, of which only the association with *Schaalia* remained significant after correction for multiple comparisons (R_s_ = .2830; *p*_adj_ = .0001).

**Table 1:** Descriptive statistics of the relative abundance (percentage) of the 23 most common genera detected across all participants, which make up about 85% of the total bacterial abundance in saliva.

Genera	Min	Max	Median	Mean	SD
*Prevotella*	0.966	38.079	13.439	15.042	8.327
*Streptococcus*	3.325	34.859	10.320	11.635	5.828
*Neisseria*	0.072	34.532	9.466	10.550	7.465
*Veillonella*	0.199	22.330	8.555	9.343	3.916
*Haemophilus*	0.309	21.072	7.403	7.705	3.901
*Rothia*	0.804	30.728	5.387	6.384	4.254
*Porphyromonas*	0.113	18.421	2.961	4.012	3.467
*Candidatus Saccharibacteria unclassified*	0.001	16.088	2.881	3.878	3.342
*Actinomyces*	0.185	15.337	2.776	3.432	2.711
*Gemella*	0.286	16.928	2.377	3.159	2.564
*Fusobacterium*	0.045	9.232	2.365	2.659	1.603
*Bacteroidaceae unclassified*	0.007	9.074	1.098	1.757	1.742
*Campylobacter*	0.129	4.876	0.890	1.037	0.685
*Granulicatella*	0.015	5.784	0.686	0.947	0.932
*Lancefieldella*	0013	3.801	0.662	0.924	0.848
*Capnocytophaga*	0.036	4.195	0.650	0.857	0.697
*Lautropia*	0.012	6.622	0.457	0.840	1.063
*Oribacterium*	0.017	3.166	0.599	0.754	0.545
*Leptotrichia*	0.045	3.053	0.635	0.748	0.512
*Corynebacterium*	0.003	8.553	0.213	0.347	0.663
*Mogibacterium*	0.001	1.321	0.225	0.293	0.263
*Lachnoanaerobaculum*	0.022	1.544	0.177	0.245	0.226
GGB1843*	0.010	1.033	0.179	0.218	0.145

*Note.* *GGB1843 represents a Genus-level Genome Bin (GGB) defined within the MetaPhlAn profiling database as a distinct genus-level lineage within the family *Bacteroidaceae.*

### Bacterial communities within co-occurrence networks

An analysis of microbial co-occurrence networks revealed that 91 of 237 genera could be combined into 12 co-abundant taxonomic groups, hereinafter referred to as Communities, each comprising 2–18 genera (Table 2; Fig 1A). The Communities’ composition and relative abundance are represented in S3 Table in S1 File. A pairwise analysis of the Community distributions based on R_s_ (Fig 1C) revealed that the first five Communities (0−4) with the highest genus richness predominantly show negative relationships between their abundance patterns. The most robust and profound association was between Community 0 (Mean abundance = 33.47; Dominant genera: *Prevotella* and *Veillonella*) and Community 2 (Mean abundance = 20.45; Dominant genera: *Neisseria* and *Haemophilus*). A high negative correlation (R_s_ = −0.804; *p*_adj_ < 0.001) indicates almost opposite abundance patterns of these Communities, suggesting that the overall pattern of core microbiome members in saliva varies significantly across individuals. Despite high inter-individual variability, the correlation analyses, WMW tests, and hierarchical clustering (Fig 1B) did not reveal a significant association between the Communities’ distribution and the participants’ demographics.

**Table 2:** Descriptive statistics of the relative abundance of 12 bacterial communities identified based on the co-occurrence/co-abundance patterns of distinct genera.

Community	Dominant Genera	No. of Genera	Relative Abundance (%)			
			Min	Max	Mean	SD
Community 0	*Prevotella; Veillonella*	18	1.667	70.204	33.474	14.878
Community 1	*Ca.Nanosynsaccharibacterium; Bacteroides*	18	0.005	8.414	0.782	1.189
Community 2	*Neisseria; Haemophilus*	10	1.269	49.137	20.447	10.390
Community 3	*Ca. Saccharibacteria unclassified; Clostridiales Fam XIII Incetae Sedis unclassified*	11	0.043	18.809	5.639	4.100
Community 4	*Streptococcus; Rothia*	9	8.581	58.448	24.530	9.713
Community 5	*Porphyromonas; Fusobacterium*	7	0.487	31.240	9.055	5.909
Community 6	*Leptotrichia*	5	0.078	9.153	1.359	1.155
Community 7	*Filifactor*	4	0.000	0.816	0.038	0.108
Community 8	*Kytococcus*	3	0.000	0.387	0.032	0.063
Community 9	*Aggregatibacter*	2	0.000	7.427	1.233	1.249
Community 10	*Lautropia*	2	0.015	7.227	1.072	1.187
Community 11	*Ottowia*	2	0.000	0.574	0.020	0.057
Other Genera		146	19.096	24.322	2.320	2.473

**Fig 1:**
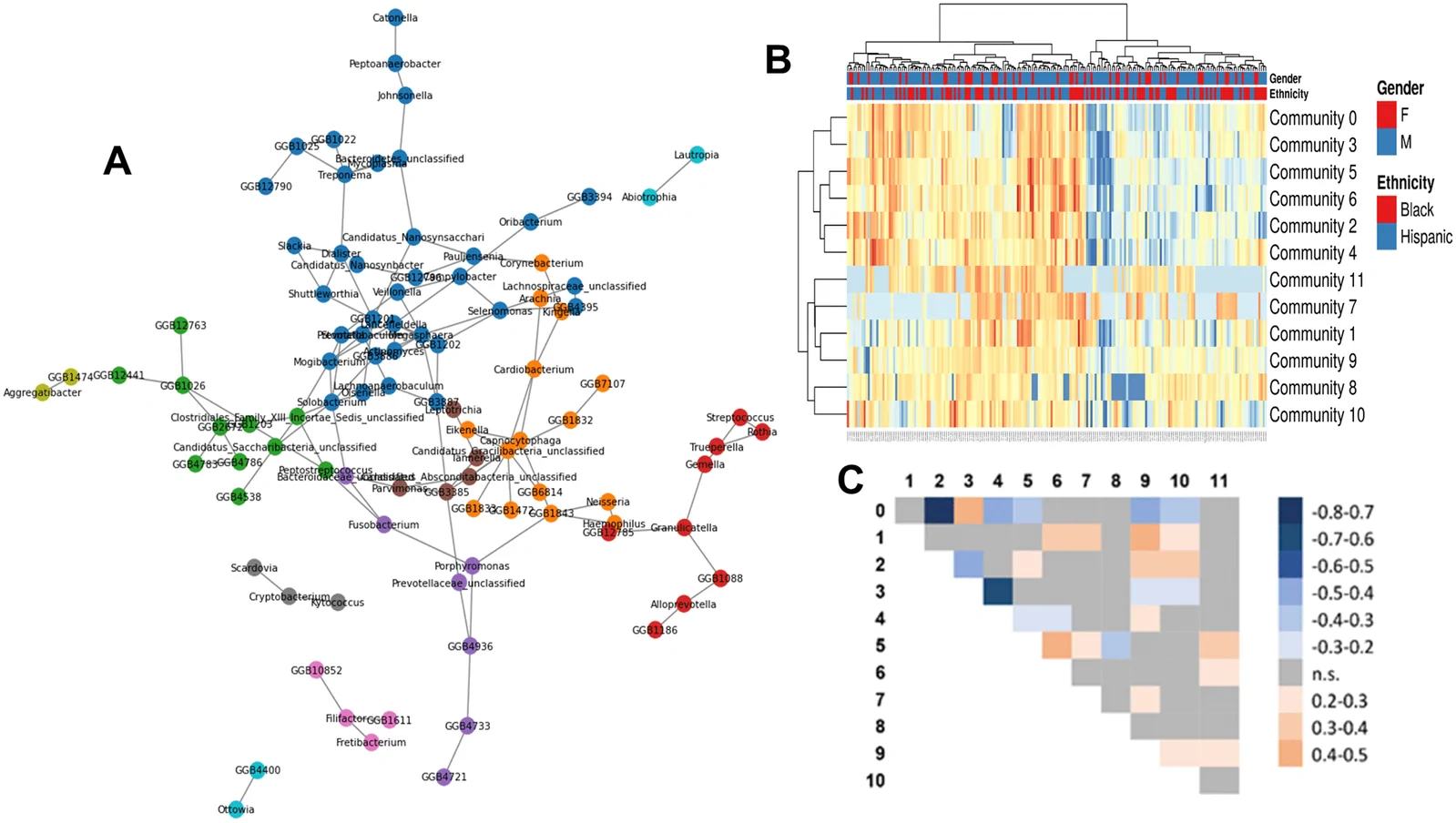
The salivary bacterial communities defined through co-occurrence/co-abundance networks. A: The network core substructures depict 12 distinct communities of bacterial genera; descriptive statistics of the genera’s relative abundance are shown in Table 2. B: The results of hierarchical clustering based on data of the communities’ relative abundances (rows) across 200 individuals (columns) were performed using Ward’s clustering algorithm and Manhattan distances. The clustering shows no impact of the participants’ demographics on the distribution of microbiome Communities: individuals of the same sex and ethnicity did not tend to cluster together. C: The heatmap represents a correlation matrix of the communities’ relative abundances. The legend depicts the color scale of intervals of Spearman’s correlation coefficients significant at a p_adj_ < 0.05; nonsignificant correlations are marked in gray.

### SES and microbiome

The association between SES and the microbiome was explored using a set of lasso-regressions with PC_SES_ (for 11 SES indicators presented in S4 Table in S1 File) as predictors of the microbiome α-diversity, the Community abundances, and the first three PC_MB_ that explain 40% of the total abundance variability of 83 most common genera (S5 Table in S1 File). There was no significant association between SES and α-diversity, and the main patterns of variability in taxa abundances identified by PC_MB_. However, the abundances of most Communities (7 of 12 identified) showed a small but significant (*p* < .05) association with SES; the determination coefficient (R^2^) ranged from 0.02 to 0.04. Notably, similar results regarding the effect of SES on compositional measures of the gut microbiome were reported in a recent study in a representative (N = 825) multi-ethnic cohort of adults, where R^2^ varied from 0.004 to 0.013 for various SES indicators [29].

### Microbiome and cognitive functioning

The association between the microbiome α-diversity and cognitive functioning was explored using univariate linear regressions with Richness and Simpson’s D as predictors for the WISC scores. There were no significant associations between WISC scores and Simpson’s D, whereas Richness was negatively associated with FRI (β = −0.1487; 95% CI = −0.2873–0.0112; *p* = .036) and WMI (β = −0.1677; 95% CI = −0.3062–0.0292; *p* = .018) subtests, and with both composite scores, NVIQ (β = −0.1567; 95% CI = −0.2951–0.0182; *p* = .027) and FSIQ (β = −0.1474; 95% CI = −0.2860–0.0088; *p* = .038). Genus-specific analysis was performed using Spearman’s correlations between the genus and Community abundances and WISC scores (S6 Table in S1 File); a visual summary of the results is shown in Fig 2. Regarding distinct taxa, 13 of 83 genera were significantly associated with at least two WISC scores. At the Community level, three of the 12 Communities were related to two or more WISC scores. In particular, the abundance of Community 2 (Dominant genera: *Neisseria* and *Haemophilus*) was positively associated, while the abundance of Community 1 (*Ca. Nanosynsaccharibacterium* and *Bacteroides*) and Community 7 (*Filifactor*) was negatively related to WISC scores.

**Fig 2:**
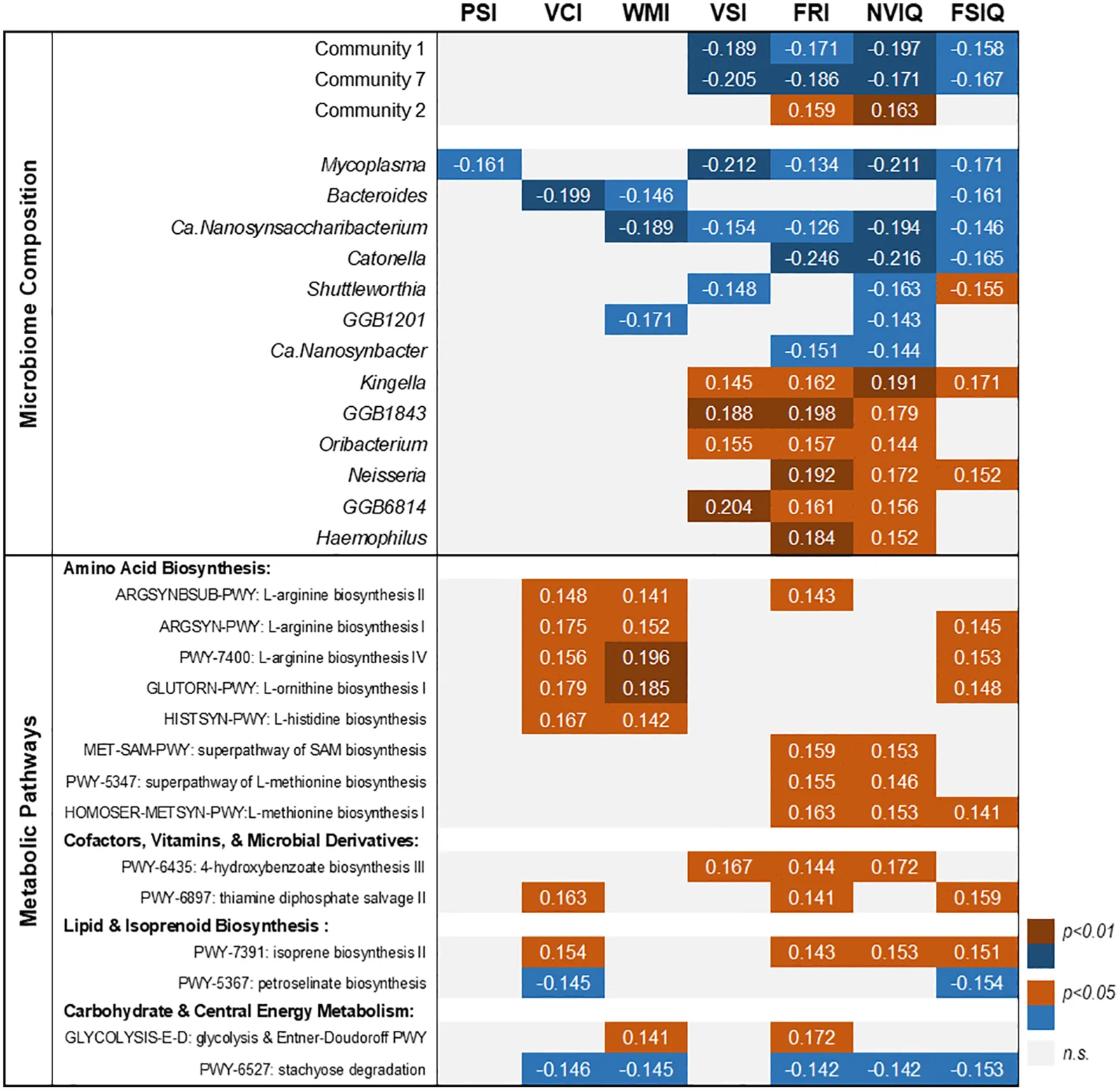
A summary of the association analysis between the microbiome and cognitive performance. The heatmap depicts Spearman’s correlation coefficients between the abundances of distinct genera, bacterial communities, and metabolic pathways in oral microbiota and cognitive abilities as per WISC scores: Processing Speed (PSI), Verbal Comprehension (VCI), Working Memory (WMI), Visual-Spatial (VSI), Fluid Reasoning (FRI), Nonverbal IQ (NVIQ), and Full-Scale IQ (FSIQ) indexes. Positive and negative correlations are marked in brown and blue, respectively. Color saturation indicates the significance level of the correlation.

Metagenomic functional annotation revealed 485 metabolic pathways across 200 salivary samples, with 320 pathways detected in at least 50% of individuals. The relative abundances of these pathways are detailed in Supplementary Table S9 in S1 File. Based on Spearman correlations, several metabolic pathways showed weak-to-moderate, yet significant, associations with at least two WISC scores, as visualized in Fig 2. Notably, a number of positive associations were observed between cognitive function scores and the abundance of pathways related to the biosynthesis of amino acids and their derivatives, such as arginine, ornithine, methionine, and S-adenosylmethionine (SAM). Also, higher cognitive scores were positively associated with the biosynthesis of 4-hydroxybenzoate—an aromatic compound vital for ubiquinone (coenzyme Q) synthesis—thiamin diphosphate salvage II involved in vitamin B1 recycling, and isoprene biosynthesis II involved in lipid building. In contrast, pathways such as petroselinate biosynthesis, which contribute to fatty acid and lipid production, and stachyose degradation, which reflects the catabolic breakdown of complex carbohydrate sugars, showed negative correlations with WISC scores (Fig 2). Together, these patterns suggest that specific metabolic functions of the oral microbiome may be differentially linked to cognitive performance.

The overall association between the microbiome and cognitive functioning was examined using lasso regressions with the leading profiles of the genera abundance variability (first 19 PC_MB_; Eigenvalue > 1.2; S5 Table in S1 File) as predictors of WISC scores (S7 Table in S1 File). The primary results are shown in Table 3 and Fig 3; the effect size varied across different cognitive indicators. In particular, there was no effect on the Verbal Comprehension (VCI); for most WISC scores (WMI, PSI, VSI, FSIQ), R^2^ varied from 0.04 to 0.06; the strongest association was with NVIQ (R^2^ = 0.109) and FRI (R^2^ = 0.140). Notably, the estimate obtained for FSIQ (R = 0.25, F = 6.55, *p* = .002) was similar to that reported in a recent study [26] of the association between the gut microbiome and FSIQ among school-age children from the Israeli Arab population (R  =  0.24, F  =  9.73, *p*  =  0.014).

**Table 3:** Primary results of regression analysis of the relationship between SES and microbiome measures and cognitive outcomes.

Predictor	Outcome*	Regression Results				
		R	R^2^_adj_	Df	F	*p*
Microbiome^†^	WISC:FRI	0.397	0.140	4;195	9.10	<0.001
	WISC:PSI	0.262	0.059	2;197	7.26	0.001
	WISC:VCI	0.000	0.000	0;199		
	WISC:VSI	0.251	0.053	2;197	6.62	0.002
	WISC:WMI	0.201	0.035	1;198	8.29	0.004
	WISC:NVIQ	0.367	0.109	3;196	8.68	<0.001
	WISC:FSIQ	0.250	0.053	2;197	6.55	0.002
SES^‡^	WISC:FRI	0.182	0.028	1;198	6.82	0.010
	WISC:PSI	0.245	0.055	1;198	12.65	<0.001
	WISC:VCI	0.275	0.071	1;198	16.20	<0.001
	WISC:VSI	0.156	0.019	1;198	4.95	0.027
	WISC:WMI	0.209	0.039	1;198	9.01	0.003
	WISC:NVIQ	0.211	0.042	1;198	9.72	0.002
	WISC:FSIQ	0.276	0.072	1;198	16.38	<0.001
SES & Microbiome	WISC:FRI	0.396	0.139	4;195	9.06	<0.001
	WISC:PSI	0.330	0.100	2;197	12.07	<0.001
	WISC:VCI	0.309	0.086	2;197	10.39	<0.001
	WISC:VSI	0.286	0.068	3;196	5.84	0.001
	WISC:WMI	0.270	0.064	2;197	7.77	0.001
	WISC:NVIQ	0.368	0.122	3;196	10.24	<0.001
	WISC:FSIQ	0.339	0.106	2;197	12.83	<0.001

*Note.* Outcome* – cognitive functioning measures as per WISC scores: Processing Speed (PSI), Verbal Comprehension (VCI), Working Memory (WMI), Visual-Spatial (VSI), Fluid Reasoning (FRI), Nonverbal IQ (NVIQ), and Full-Scale IQ (FSIQ) indexes. Microbiome^†^ – the leading patterns of the genera’s abundance variability described by the first 19 PC_MB_ (see Methods, and S4 Table in S1 File). SES^‡^ – overall SES profiles (PC_SES_) generated based on the PC analysis of the variability of 11 SES indicators (see Methods and S2 Table in S1 File).

**Fig 3:**
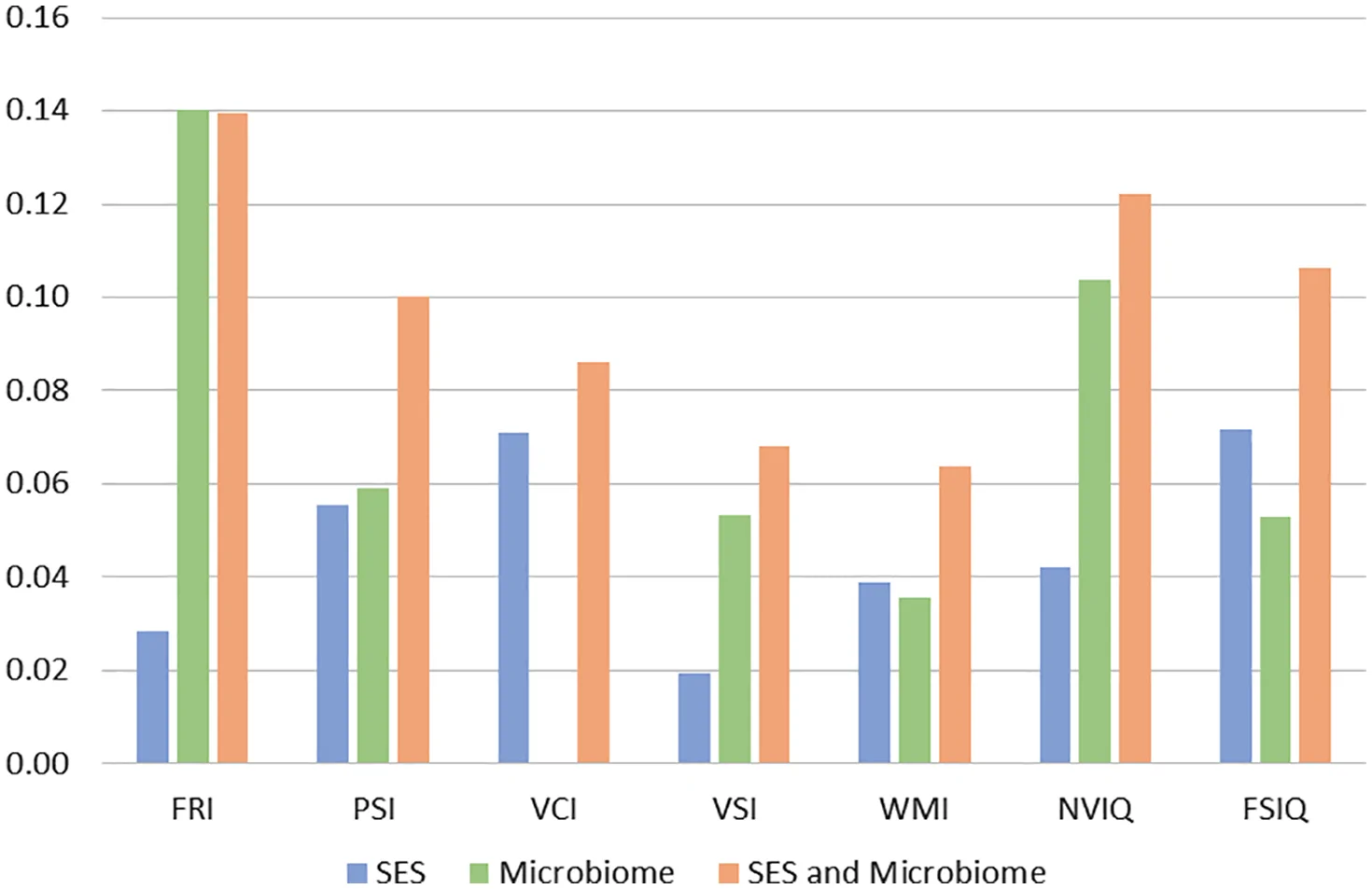
A summary of the association analysis between the microbiome, SES, and cognitive performance. The histogram shows the effect sizes (R2) from regression models predicting WISC scores based on PCs of microbiome composition and SES.

### Joint association of SES and microbiome with cognitive performance

To explore the unique and joint associations of the microbiome and SES with cognitive performance, we included both PC_SES_ and PC_MB_ as predictors of WISC scores (S7 Table in S1 File). The primary results of the regression analysis are shown in Table 3 and Fig 3; the correlations of most cognitive indicators with the microbiota patterns appear to be comparable and, for FRI and NVIQ, even more robust than those with SES (z = 2.34, *p* = .010 and z = 1.71, *p* = .044, for FRI and NVIQ, respectively). On the contrary, VCI showed more substantial association with SES than with the microbiome (z = 2.80, *p* = .003).

Table 3 and Fig 3 show that SES and microbiome exert differential contributions to predicting WISC scores. Importantly, including both SES and microbiome characteristics as predictors of cognitive performance significantly increased the effect sizes of the regression models across all cognitive domains reflected in WISC scores. In particular, the mean effect size from combined models (SES and microbiome; R^2^ = 0.098; SD = 0.028) exceeds 1.5–2 times those derived only with the microbiome (R^2^ = 0.064; SD = 0.031; t(df) = 2.15(12), *p* = .052) and only with SES (R^2^ = 0.047; SD = 0.020; t(df) = 3.92(12), *p* = .002). Such an additive effect suggests that combining conventional SES indicators with microbiome characteristics may better reflect the complex interplay of factors that collectively contribute to cognitive development and function.

## Discussion

In this study, we characterized the diversity and composition of the oral microbiome using a metagenomics approach to salivary DNA sequencing data from an ethnically diverse sample of 200 children. Consistent with prior work [28], we observed substantial inter-individual variability in α-diversity and the distribution of distinct microbial genera and their co-existing core communities. Notably, this variability did not differ across racial or ethnic groups, contrary to earlier reports [30]. This absence of group differences can be partially explained by similar lifestyles—an important factor shaping microbiota diversity and composition [5–7]—as the participants resided in a relatively homogeneous urban environment in New York City (NYC). Similar patterns of convergent microbial profiles driven by urbanization have been documented in transcontinental comparisons of American and Chinese populations [31]. We also found a significant positive correlation between genus richness and age. Although this association did not survive correction for multiple comparisons, the large number of nominally significant relationships across more than 20% of common genera suggests a meaningful developmental pattern.

Our findings also contribute to the growing literature on associations between the oral microbiome and SES. Research on how SES shapes the microbiome remains limited, though existing evidence indicates that SES indicators such as family income and education influence oral-microbiome composition in ways comparable to race/ethnicity, diet, and oral health [32]. In this study, SES was conceptualized as a cumulative construct incorporating family income, parental educational attainment, occupational prestige, residence type, and neighborhood characteristics. We observed that variability in SES indicators was reflected in the distribution of several co-existing microbial communities across individual microbiomes.

This study also provides new insights into the relationship between the oral microbiome and cognitive functioning. Both overall microbiome diversity and the abundance of specific genera were associated with children’s global and domain-specific cognitive performance. In particular, the strongest positive correlations were observed for the abundances of Kingella, Neisseria, and Haemophilus, whereas the abundances of Catonella, Mycoplasma, Bacteroides, *Ca. Saccharibacteria*, and *Treponema* showed negative associations. Notably, the latter three genera have previously been associated with cognitive decline in older adults, with increased abundance associated with poorer cognition in a study of the subgingival microbiome [23].

We observed that genus richness was negatively associated with WISC scores, whereas Simpson’s D, which incorporates both richness and evenness, showed no significant association. This pattern suggests that lower cognitive scores may be associated with the emergence of rare taxa in the oral microbiota. Interestingly, this contrasts with findings from the gut microbiome, where higher α-diversity has been associated with higher FSIQ scores [26]. In older adults, oral microbiome α-diversity has been associated with teeth count rather than cognitive performance [23]. Together, these discrepancies suggest that the direction and strength of microbiota-cognition associations may vary across developmental stages and microbial ecosystems (i.e., oral vs. gut).

Little is known about the oral microbiota’s metabolites and pathways related to cognitive functioning, as findings have predominantly originated from gut microbiome studies, and oral and gut microbiotas differ in their resident bacteria; consequently, their metabolomic profiles also vary [33]. Our metagenomic functional annotation, followed by an association analysis with cognition, revealed several associations between cognitive performance and amino acid metabolic pathways, including those related to L-arginine, its downstream metabolite L-ornithine, and methionine biosynthesis. L-arginine is a vital amino acid for cognitive health, and disruptions in its homeostasis parallel age-related cognitive decline [34]. Methionine and its derivative, S-adenosylmethionine (SAM), regulate primary epigenetic machinery via DNA methylation. The positive correlation observed here aligns with evidence that adequate SAM levels support neurogenesis, maintain myelin integrity, and protect against neurodegenerative processes [35–37].

A central aim of this study was to compare the contributions of the microbiome and SES to cognitive functioning. SES reflects access to social and economic resources that shape cognitive development across the lifespan. The microbiome, in turn, is influenced by both internal (e.g., genetics, nutritional intake, immune system function, and other host-microbe interactions) and external (e.g., lifestyle and environmental exposures) factors, which are not independent of each other. As such, the microbiome can be viewed both as a product of these influences and as an indicator of their cumulative effects. Given its emerging role in brain health and cognitive function we hypothesized that SES and microbiome would serve as complementary predictors of cognitive development with comparable effect sizes. Our results support this hypothesis. Associations between summative and domain-specific cognitive performance, as assessed by the WISC, and the oral microbiota were similar in magnitude to those between the WISC and SES. Moreover, including both SES and microbiome in a multiple regression model nearly doubles the explained variance in cognitive performance (R^2^ = 0.098) compared with models including only the microbiome (R^2^ = 0.064) or SES (R^2^ = 0.047). These results suggest that SES and microbiome function as semi-independent predictors of cognitive performance. This perspective resonates with theories linking the Flynn effect [38] to SES and nutrition [39] and may help resolve long-standing puzzles in the cognitive-development literature [40].

Several limitations of this study should be noted. First, our SES indicators did not include dietary exposures and oral health—two closely intertwined factors that shape the microbiome through a bidirectional cycle. Poor oral health can restrict dietary choices, while inadequate nutrition can exacerbate oral diseases; both may substantially affect microbiome composition and developmental processes and may substantially influence a child’s cognitive function. Future work should incorporate these variables as key mediators and modifiable risk factors. Second, the cross-sectional design precludes conclusions about causality or developmental trajectories. Longitudinal studies are needed to clarify the directionality and persistence of associations between the microbiome and cognitive performance. Third, our taxonomic profiling and association analyses were intentionally restricted to the genus level. While this approach was adopted as a conservative strategy to ensure robust classification, mitigate severe zero-inflation, and reduce dimensionality to maintain statistical power, it inherently limits the taxonomic resolution of our findings regarding specific microbial species. Fourth, while a larger sample size obtained by incorporating public datasets would be highly desirable to maximize statistical power, the moderate size of our current cohort was a necessary trade-off. The unique and multi-faceted nature of our dataset—which concurrently captures individual-level data on the oral microbiome, SES, and normative cognitive functioning—made the integration of external data unfeasible without introducing unmanageable confounding factors. Fifth, the sample was limited to Hispanic and Black American children from the greater NYC area, which may constrain the generalizability of the findings, particularly regarding associations between cognitive performance and specific microbial taxa and their communities, as such relationships may vary across regions. Finally, interpretation is limited by the relatively small body of existing research on the interplay among the oral microbiome, SES, and cognition.

Despite these limitations, this study has notable strengths, including robust sampling of children from a well-characterized, ethnically diverse cohort, with intentional oversampling of groups historically underrepresented in genetic research, particularly in genomic sequencing. The shared geographic context of NYC also minimizes confounding related to regional environmental variation.

In conclusion, our findings support a framework in which SES and the microbiome function as complementary, equipollent predictors of cognitive performance. Considering both factors provides a more comprehensive understanding of the multifaceted influences on cognitive development and function. Future research integrating social and biological determinants of health may inform interventions aimed at promoting brain and cognitive health and reducing SES-related disparities, potentially by addressing both SES inequalities and microbial well-being.

## Methods

### Sample

The sample included children and youth participating in the Healthy Brain Network (HBN) initiative, an ongoing project administered by the Child Mind Institute (https://healthybrainnetwork.org/). The HBN focused on creating and sharing a bank of data about psychiatric, behavioral, and cognitive phenotypes, as well as brain-imaging and genetic characteristics, of a community sample of children and adolescents residing in the greater New York City area. The HBN data collection procedures are described in details elsewhere [41]; the datasets are openly accessible without additional ethical or administrative clearances. In this study, we utilized datasets on participants’ demographics and behavior from the publicly available HBN curated data repository (https://fcon_1000.projects.nitrc.org/indi/cmi_healthy_brain_network/; Data Release 9.0, accessed 06/03/2023).

Given prior literature on the association between microbiome and cognition [25,26], which reports effect sizes (R^2^) ranging from 0.02 to 0.14 (mean R^2^ = 0.06), the desired sample size was estimated at n = 197 (at 1-β = 80% and α ≤ .05). A key assumption underlying this analysis was the relative homogeneity of the sample. To support this assumption, we structured the sample by excluding non-Hispanic White participants, given the well-documented IQ gap between this group and other racial/ethnic groups—differences that have been attributed, in part, to SES disparities. This gap has been observed since the early days of psychometric testing in the United States, although its magnitude has declined over time [42].

In this study, bio-behavioral data from 200 Hispanic and African American HBN participants were used (S8 Table in S1 File); the participants were selected based on the parental self-stated ethnicity. The age ranged from 6 to 16 years (M = 10.16, SD = 2.46); 25% of the children (n = 51) were female at birth. Descriptive statistics for demographics across the four participant groups defined by sex and ethnicity are provided in Table 4. There were no significant differences in age distribution across sex and ethnic groups (*𝜒*^2^(3) = 3.219, *p* = 0.359) and in the sex ratio between the ethnic groups (*𝜒*^2^ = 1.306, *p* = 0.253).

**Table 4:** Descriptive statistics of demographic characteristics, WISC scores, and SES indicators stratified by sex and ethnicity.

	African Americans		Hispanic Americans	
	Males N = 68	Females N = 28	Males N = 81	Females N = 23
Age Range	6.03-16.37	6.7-15.18	6.05-15.23	6.52-15.28
Age (Mean; SD)	10.33 (2.51)	10.37 (2.34)	9.88 (2.36)	10.41 (2.67)
Born in the US (n; %)	67 (98.5%)	28 (100%)	80 (98.8%)	22 (95.7%)
*WISC scores:*				
Fluid Reasoning, FRI (Mean; SD)	93.06 (18.07)	88.75 (12.21)	93.14 (15.50)	91.48 (16.02)
Processing Speed, PSI (Mean; SD)	87.52 (14.88)	93.25 (14.77)	88.20 (16.54)	88.91 (13.48)
Verbal Comprehension, VCI (Mean; SD)	93.30 (15.98)	95.18 (14.67)	92.79 (17.03)	93.17 (16.40)
Visual-Spatial, VSI (Mean; SD)	89.41 (15.18)	88.25 (13.27)	94.66 (15.22)	91.48 (14.38)
Working Memory, WMI (Mean; SD)	90.22 (15.39)	88.00 (13.42)	89.09 (14.49)	93.57 (10.87)
Nonverbal IQ, NVIQ (Mean; SD)	87.81 (16.24)	86.71 (12.30)	89.68 (15.00)	89.17 (14.83)
Full-Scale IQ, FSIQ (Mean; SD)	88.81 (15.77)	86.93 (12.17)	88.76 (15.09)	88.61 (14.42)
*SES indicators:*				
BSMSS: Parental education (Mean; SD)	16.28 (3.39)	15.91 (3.73)	15.57 (3.34)	13.63 (4.93)
BSMSS: Parental occupation (Mean; SD)	29.03 (11.73)	26.61 (13.20)	24.69 (11.59)	20.98 (15.11)
PhenX: Neighborhood safety (Mean; SD)	6.91 (2.94)	7.50 (2.98)	6.78 (2.79)	7.96 (2.87)
FSQ: Household income (Mean; SD)	6.30 (3.13)	5.54 (3.53)	5.95 (2.95)	4.87 (3.86)
FSQ: Other financial support (# of sources Mean; SD)	0.33 (0.64)	0.68 (0.94)	0.44 (0.84)	0.91 (1.20)
FSQ: Residence own or rent (n, % of own)	24 (35.29%)	12 (42.86%)	29 (35.80%)	7 (30.43%)
FSQ: Residence type (Mean; SD)	1.42 (1.63)	1.43 (1.75)	1.17 (1.70)	2.35 (1.99)
FSQ: Parent 1 employed (unemployed n; %)	10 (14.71)	5 (17.86)	13 (16.05)	6 (26.10)
FSQ: Parent 2 employed (unemployed n; %)	22 (32.35)	10 (35.71)	22 (27.16)	8 (34.78)
FSQ: People in the home (n; SD)	4.09 (1.65)	3.64 (1.31)	4.09 (1.30)	4.22 (1.48)
FSQ: Health insurance availability (available n; %)	67 (98.5%)	26 (92.9%)	77 (95.1%)	23 (100%)

*Note.* The SES descriptives are presented as percentages for binary scales and as mean and SD for numeric scales; for details on the scores, please see the Phenotypic Assessment Protocol on the HBN scientific data portal (https://healthybrainnetwork.org/).

### Ethical statement

All methods were carried out in accordance with the ethical standards of the institutional and national research committees, and with the 1964 Helsinki Declaration and its later amendments or comparable ethical standards. All protocols and procedures of this study were approved by the University of Houston Institutional Review Board (IRB ID: STUDY00000093; October 30, 2018). All participants provided written informed consent; for participants younger than 18, written consent was obtained from their legal guardians.

### Cognitive assessments

The Wechsler Intelligence Scale for Children, WISC-V, was used, which consists of five subtest scores and two composite intelligence scores—NVIQ and FSIQ [43]. Individual scores are presented in S8 Table in S1 File; descriptive statistics for WISC scores across the four sex/ethnic participant groups are shown in Table 4. No significant group differences were revealed with the Kruskal-Wallis tests (df = 3; N = 200): *H*_FRI_  = 2.39, *p* = 0.495; *H*_PSI_ = 2.02, *p* = 0.568; *H*_VCI_ = 0.641, *p* = 0.887; *H*_VSI_ = 3.90, *p* = 0.272; *H*_WMI_ = 2.79, *p* = 0.425; *H*_NVIQ_ = 0.88, *p* = 0.830; *H*_FSIQ_ = 0.724, *p* = 0.868). There was a broad range of variability in cognitive functioning (FSIQ Min = 53, Max = 124, Mode = 79, Median = 87). The sample performed in the normative range of 85–115; the values were 88.56 ± 15.02 and 88.51 ± 14.80 for NVIQ and FSIQ, respectively. Pairwise correlations between WISC scores varied from 0.3345 (*p* < .001) between WMI and VSI to 0.9066 (*p* < .001) between NVIQ and FSIQ (Mean R = 0.6236; SD = 0.1677).

### Socioeconomic status (SES) indicators

The SES and child’s home environment were assessed using the Barratt Simplified Measure of Social Status, BSMSS [44]; the Financial Support Questionnaire, FSQ [45]; and the Phenotypes and Exposures, PhenX Toolkit: Neighborhood Safety measures [46]. These assessments’ numeric scores characterized the parents’ educational attainment, employment and occupational prestige, the presence of medical insurance, residence type and neighborhood, household income, and the number of people cohabiting in the households. The SES indicators are presented in S8 Table in S1 File; a summary of descriptive statistics is provided in Table 4. The global SES profiles were generated as the principal components (PC) of the SES indicators’ variability (S4 Table in S1 File); these PC_SES_ were included in the SES-microbiome-cognition statistical modeling.

### Microbiome profiling

Saliva specimens were collected using the OG-500 Oragene-DNA collection kit (DNA Genotek, Ottawa, ON, Canada), which contains a preservative that stabilizes salivary DNA content, including that derived from the microbiota [47]. Participants were asked to avoid eating, drinking, smoking, or chewing gum for 30 minutes prior to saliva collection. Saliva samples (2 mL) were collected by spitting into the OG-500 collection tubes. DNA was isolated using the Oragene/prepIT•L2P method as per the manufacturer’s protocol that includes incubating saliva samples (50°C, 2 hr) to release DNA, adding prepIT•L2P reagent to precipitate impurities, and centrifuging to isolate DNA, followed by ethanol precipitation for purification. DNA sequencing was performed at the Human Genome Sequencing Center of Baylor College of Medicine using the Illumina TruSeq DNA PCR-Free protocol for library preparation and the Illumina NovaSeq 6000 sequencing platform with a target sequencing depth of 30X human genome coverage. Raw reads were mapped to the GRCh38 (human) genome using the Burrows-Wheeler Alignment tool, BWA (https://github.com/lh3/bwa), followed by alignment processing with the Sequence Alignment/Map tools, SAMtools v.1.18 (https://github.com/samtools; accessed 01/09/2023). The reads unmapped to the human genome were used for the metagenome taxonomic profiling with MetaPhlAn v. 4.0.6 (https://github.com/biobakery/MetaPhlAn; accessed 01/01/2024), using default parameters and the vOct22 database. The metagenomic functional profiling was performed using the HMP Unified Metabolic Analysis Network (HUMAnN 3.0; https://github.com/biobakery/HUMAnN; accessed 01/01/2024).

### Statistical analysis

Statistical analysis was conducted on three microbiome measures: α-diversity, individual taxa abundance, and microbial community abundance. The primary analysis was performed at the genus level, as this is the most refined taxonomic level for predicting complex traits [48]. The data on the relative abundances of genera and communities are presented in S1 and S3 Tables in S1 File, respectively. Indices of α-diversity were estimated as Richness (the number of distinct genera per an individual’s microbiome) and Simpson’s reciprocal index of diversity, Simpson’s D—a function of both richness and evenness [49]. The microbial communities were identified through a co-occurrence network analysis using the COmpositional Zero-Inflated Network Estimation (COZINE) method [50]. To evaluate network robustness, 100 bootstrap runs with COZINE were performed; the stability of an edge was defined as the fraction of bootstrap samples in which the edge was present in the resulting networks.

Association analysis was conducted between the cognitive and the microbiome indices using Spearman’s rank-order correlations (R_s_) and univariate and multivariate regression. Benjamini-Hochberg adjusted *p*-values were used whenever appropriate to control for multiple testing. Before the analysis, the genera with a count of zero in at least 50% of samples were removed to minimize the influence of rare taxa and spurious results. After filtering, 83 of 237 (35%) genera remained (S1 Table in S1 File). Second, relative abundance indices were adjusted for age, sex, and ethnicity; the standardized residuals were used in further analyses. A summary microbiome profile was generated based on the PC of the 83 genera’s abundance variability (S5 Table in S1 File); these PC_MB_ were included in the microbiome-cognition statistical modeling. The associations between the microbiome, SES, and cognitive functioning profiles reconstructed based on PCA were examined using lasso regression with the *glmnet* package (https://glmnet.stanford.edu/).

## Supporting information

S1 File Supplementary Tables S1-S9. (XLSX)

## References

[1] Langensee L, Rumetshofer T, Mårtensson J. Interplay of socioeconomic status, cognition, and school performance in the ABCD sample. NPJ Sci Learn. 2024;9(1):17. doi: 10.1038/s41539-024-00233-x 38467686 PMC10928106

[2] Hackman DA, Farah MJ, Meaney MJ. Socioeconomic status and the brain: mechanistic insights from human and animal research. Nat Rev Neurosci. 2010;11(9):651–9. doi: 10.1038/nrn2897 20725096 PMC2950073

[3] Kim EJ, Kim JJ. Neurocognitive effects of stress: a metaparadigm perspective. Mol Psychiatry. 2023;28(7):2750–63. doi: 10.1038/s41380-023-01986-4 36759545 PMC9909677

[4] Larnyo E, Dai B, Nutakor JA, Ampon-Wireko S, Larnyo A, Appiah R. Examining the impact of socioeconomic status, demographic characteristics, lifestyle and other risk factors on adults’ cognitive functioning in developing countries: an analysis of five selected WHO SAGE Wave 1 Countries. Int J Equity Health. 2022;21(1):31. doi: 10.1186/s12939-022-01622-7 35216605 PMC8876754

[5] Lindheim L, Bashir M, Münzker J, Trummer C, Zachhuber V, Pieber TR, et al. The Salivary Microbiome in Polycystic Ovary Syndrome (PCOS) and its association with disease-related parameters: a pilot study. Front Microbiol. 2016;7:1270. doi: 10.3389/fmicb.2016.01270 27610099 PMC4996828

[6] Wang K, Lu W, Tu Q, Ge Y, He J, Zhou Y, et al. Preliminary analysis of salivary microbiome and their potential roles in oral lichen planus. Sci Rep. 2016;6:22943. doi: 10.1038/srep22943 26961389 PMC4785528

[7] Shaw L, Ribeiro ALR, Levine AP, Pontikos N, Balloux F, Segal AW, et al. The human salivary microbiome is shaped by shared environment rather than genetics: evidence from a large family of closely related individuals. mBio. 2017;8(5):e01237-17. doi: 10.1128/mBio.01237-17 28900019 PMC5596345

[8] Liu L, Huh JR, Shah K. Microbiota and the gut-brain-axis: Implications for new therapeutic design in the CNS. EBioMedicine. 2022;77:103908. doi: 10.1016/j.ebiom.2022.103908 35255456 PMC8897630

[9] Chen M, Ruan G, Chen L, Ying S, Li G, Xu F, et al. Neurotransmitter and intestinal interactions: focus on the microbiota-gut-brain axis in irritable bowel syndrome. Front Endocrinol (Lausanne). 2022;13:817100. doi: 10.3389/fendo.2022.817100 35250873 PMC8888441

[10] Puricelli C, Rolla R, Gigliotti L, Boggio E, Beltrami E, Dianzani U, et al. The gut-brain-immune axis in autism spectrum disorders: a state-of-art report. Front Psychiatry. 2022;12:755171. doi: 10.3389/fpsyt.2021.755171 35185631 PMC8850385

[11] Fernández J, Redondo-Blanco S, Gutiérrez-del-Río I, Miguélez EM, Villar CJ, Lombó F. Colon microbiota fermentation of dietary prebiotics towards short-chain fatty acids and their roles as anti-inflammatory and antitumour agents: A review. Journal of Functional Foods. 2016;25:511–22. doi: 10.1016/j.jff.2016.06.032

[12] Yang Y, Tian J, Yang B. Targeting gut microbiome: A novel and potential therapy for autism. Life Sci. 2018;194:111–9. doi: 10.1016/j.lfs.2017.12.027 29277311

[13] Ma Q, Xing C, Long W, Wang HY, Liu Q, Wang R-F. Impact of microbiota on central nervous system and neurological diseases: the gut-brain axis. J Neuroinflammation. 2019;16(1):53. doi: 10.1186/s12974-019-1434-3 30823925 PMC6397457

[14] Morais LH, Schreiber HL 4th, Mazmanian SK. The gut microbiota-brain axis in behaviour and brain disorders. Nat Rev Microbiol. 2021;19(4):241–55. doi: 10.1038/s41579-020-00460-0 33093662

[15] Kunath BJ, Hickl O, Queirós P, Martin-Gallausiaux C, Lebrun LA, Halder R, et al. Alterations of oral microbiota and impact on the gut microbiome in type 1 diabetes mellitus revealed by integrated multi-omic analyses. Microbiome. 2022;10(1):243. 36578059 10.1186/s40168-022-01435-4 PMC9795701

[16] Kobayashi R, Ogawa Y, Hashizume-Takizawa T, Kurita-Ochiai T. Oral bacteria affect the gut microbiome and intestinal immunity. Pathog Dis. 2020;78(3):ftaa024. doi: 10.1093/femspd/ftaa024 32504490

[17] Park S-Y, Hwang B-O, Lim M, Ok S-H, Lee S-K, Chun K-S, et al. Oral-gut microbiome axis in gastrointestinal disease and cancer. Cancers (Basel). 2021;13(9):2124. doi: 10.3390/cancers13092124 33924899 PMC8125773

[18] Hajishengallis G. Periodontitis: from microbial immune subversion to systemic inflammation. Nat Rev Immunol. 2015;15(1):30–44. doi: 10.1038/nri3785 25534621 PMC4276050

[19] Li X, Kolltveit KM, Tronstad L, Olsen I. Systemic diseases caused by oral infection. Clin Microbiol Rev. 2000;13(4):547–58. doi: 10.1128/CMR.13.4.547 11023956 PMC88948

[20] Bowland GB, Weyrich LS. The oral-microbiome-brain axis and neuropsychiatric disorders: an anthropological perspective. Front Psychiatry. 2022;13:810008. doi: 10.3389/fpsyt.2022.810008 35432038 PMC9005879

[21] Qing Y, Xu L, Cui G, Sun L, Hu X, Yang X, et al. Salivary microbiome profiling reveals a dysbiotic schizophrenia-associated microbiota. NPJ Schizophr. 2021;7(1):51. doi: 10.1038/s41537-021-00180-1 34711862 PMC8553823

[22] Chen L, Cao H, Wu X, Xu X, Ji X, Wang B, et al. Effects of oral health intervention strategies on cognition and microbiota alterations in patients with mild Alzheimer’s disease: A randomized controlled trial. Geriatr Nurs. 2022;48:103–10. doi: 10.1016/j.gerinurse.2022.09.005 36155316

[23] Fogelholm N, Leskelä J, Manzoor M, Holmer J, Paju S, Hiltunen K, et al. Subgingival microbiome at different levels of cognition. J Oral Microbiol. 2023;15(1):2178765. doi: 10.1080/20002297.2023.2178765 36844899 PMC9946326

[24] Yang I, Arthur RA, Zhao L, Clark J, Hu Y, Corwin EJ, et al. The oral microbiome and inflammation in mild cognitive impairment. Exp Gerontol. 2021;147:111273. doi: 10.1016/j.exger.2021.111273 33556534

[25] Meyer K, Lulla A, Debroy K, Shikany JM, Yaffe K, Meirelles O, et al. Association of the gut microbiota with cognitive function in midlife. JAMA Netw Open. 2022;5(2):e2143941. doi: 10.1001/jamanetworkopen.2021.43941 35133436 PMC8826173

[26] Lapidot Y, Maya M, Reshef L, Cohen D, Ornoy A, Gophna U, et al. Relationships of the gut microbiome with cognitive development among healthy school-age children. Front Pediatr. 2023;11:1198792. doi: 10.3389/fped.2023.1198792 37274812 PMC10235814

[27] Lin T-Y, Wang P-Y, Lin C-Y, Hung S-C. Association of the oral microbiome with cognitive function among older adults: NHANES 2011-2012. J Nutr Health Aging. 2024;28(8):100264. doi: 10.1016/j.jnha.2024.100264 38772098 PMC12878625

[28] Ruan X, Luo J, Zhang P, Howell K. The salivary microbiome shows a high prevalence of core bacterial members yet variability across human populations. NPJ Biofilms Microbiomes. 2022;8(1):85. doi: 10.1038/s41522-022-00343-7 36266278 PMC9584946

[29] Kwak S, Usyk M, Beggs D, Choi H, Ahdoot D, Wu F, et al. Sociobiome - Individual and neighborhood socioeconomic status influence the gut microbiome in a multi-ethnic population in the US. NPJ Biofilms Microbiomes. 2024;10(1):19. doi: 10.1038/s41522-024-00491-y 38467678 PMC10928180

[30] Premaraj TS, Vella R, Chung J, Lin Q, Hunter P, Underwood K, et al. Ethnic variation of oral microbiota in children. Sci Rep. 2020;10(1):14788. doi: 10.1038/s41598-020-71422-y 32901068 PMC7478955

[31] Winglee K, Howard AG, Sha W, Gharaibeh RZ, Liu J, Jin D, et al. Recent urbanization in China is correlated with a Westernized microbiome encoding increased virulence and antibiotic resistance genes. Microbiome. 2017;5(1):121. doi: 10.1186/s40168-017-0338-7 28915922 PMC5603068

[32] Renson A, Jones HE, Beghini F, Segata N, Zolnik CP, Usyk M, et al. Sociodemographic variation in the oral microbiome. Ann Epidemiol. 2019;35:73-80.e2. doi: 10.1016/j.annepidem.2019.03.006 31151886 PMC6626698

[33] Chen L, Xu X, Wu X, Cao H, Li X, Hou Z, et al. A comparison of the composition and functions of the oral and gut microbiotas in Alzheimer’s patients. Front Cell Infect Microbiol. 2022;12:942460. doi: 10.3389/fcimb.2022.942460 36093178 PMC9448892

[34] Dobrynina L, Byrochkina A, Shamtieva K, Kremneva E, Zabitova M, Shabalina A. L-arginine and its metabolites in age-related cerebral small vessel disease with cognitive impairment. Biomolecules. 2026;16(6):914. doi: 10.3390/biom16060914 42352381 PMC13297444

[35] Zhang Y, Liu T, Pan F, Li Y, Wang D, Pang J, et al. Dietary methionine restriction alleviates cognitive impairment in alzheimer’s disease mice via sex-dependent modulation on gut microbiota and tryptophan metabolism: a multiomics analysis. J Agric Food Chem. 2025;73(2):1356–72. doi: 10.1021/acs.jafc.4c09878 39745486

[36] Alachkar A, Agrawal S, Baboldashtian M, Nuseir K, Salazar J, Agrawal A. L-methionine enhances neuroinflammation and impairs neurogenesis: Implication for Alzheimer’s disease. J Neuroimmunol. 2022;366:577843. doi: 10.1016/j.jneuroim.2022.577843 35299077 PMC13592503

[37] Baden KER, McClain H, Craig E, Gibson N, Draime JA, Chen AMH. S-Adenosylmethionine (SAMe) for central nervous system health: a systematic review. Nutrients. 2024;16(18):3148. doi: 10.3390/nu16183148 39339750 PMC11435340

[38] Trahan LH, Stuebing KK, Fletcher JM, Hiscock M. The Flynn effect: a meta-analysis. Psychol Bull. 2014;140(5):1332–60. doi: 10.1037/a0037173 24979188 PMC4152423

[39] Shakeel MD, Peterson PE. A half century of progress in US student achievement: Agency and Flynn effects, ethnic and SES differences. Educ Psychol Rev. 2022;34:1255–342.

[40] Oberleiter S, Patzl S, Fries J, Diedrich J, Voracek M, Pietschnig J. Measurement-invariant fluid anti-Flynn effects in population-representative German student samples (2012-2022). J Intell. 2024;12(1):9. 38248907 10.3390/jintelligence12010009 PMC10816863

[41] Alexander LM, Escalera J, Ai L, Andreotti C, Febre K, Mangone A, et al. An open resource for transdiagnostic research in pediatric mental health and learning disorders. Sci Data. 2017;4:170181. doi: 10.1038/sdata.2017.181 29257126 PMC5735921

[42] Dickens WT, Flynn JR. Black Americans reduce the racial IQ gap: evidence from standardization samples. Psychol Sci. 2006;17(10):913–20. doi: 10.1111/j.1467-9280.2006.01802.x 17100793

[43] Wechsler D. Administrative and scoring manual for the Wechsler Intelligence Scale for Children. 5th ed. NCS Pearson. 2014.

[44] Barratt W. The Barratt simplified measure of social status (BSMSS). Indiana State University. 2006.

[45] Mujahid MS, Diez Roux AV, Morenoff JD, Raghunathan T. Assessing the measurement properties of neighborhood scales: from psychometrics to ecometrics. Am J Epidemiol. 2007;165(8):858–67. doi: 10.1093/aje/kwm040 17329713

[46] Hamilton CM, Strader LC, Pratt JG, Maiese D, Hendershot T, Kwok RK, et al. The PhenX Toolkit: get the most from your measures. Am J Epidemiol. 2011;174(3):253–60. doi: 10.1093/aje/kwr193 21749974 PMC3141081

[47] Hasan NA, Young BA, Minard-Smith AT, Saeed K, Li H, Heizer EM, et al. Microbial community profiling of human saliva using shotgun metagenomic sequencing. PLoS One. 2014;9(5):e97699. doi: 10.1371/journal.pone.0097699 24846174 PMC4028220

[48] Song K, Zhou YH. Leveraging scheme for cross-study microbiome machine learning prediction and feature evaluations. Bioengineering (Basel). 2023;10(2):231. 36829725 10.3390/bioengineering10020231 PMC9952031

[49] Simpson EH. Measurement of Diversity. Nature. 1949;163(4148):688–688. doi: 10.1038/163688a0

[50] Ha MJ, Kim J, Galloway-Peña J, Do K-A, Peterson CB. Compositional zero-inflated network estimation for microbiome data. BMC Bioinformatics. 2020;21(Suppl 21):581. doi: 10.1186/s12859-020-03911-w 33371887 PMC7768662

