# Supplementary material for: Salivary microbiome, socioeconomic status, and cognitive performance: Triangulating the relationship
Source: PLoS One. 2026 Nov 2;21(11):e0361602. doi: 10.1371/journal.pone.0361602 (PMC13726965; doi:10.1371/journal.pone.0361602)
Supplement: Supplementary file 1 [file pone.0361602.s003.docx]

*Dear Editors and Reviewers,*

*We are grateful for your consideration of our manuscript entitled "Salivary microbiome, socioeconomic status, and cognitive performance: Triangulating the relationship." We appreciate the constructive comments on our paper. We have carefully studied each comment and made the corresponding revisions, which we hope will meet with your approval. Below, we provide our point-by-point responses to your comments.*

*Sincerely,*

*On behalf of the authors*

*Oxana Yu. Naumova, PhD*

**Journal Requirements:**

1. Please ensure that your manuscript meets PLOS ONE's style requirements, including those for file naming.

*Response:*

We have revised and formatted the manuscript in accordance with PLOS ONE's formatting guidelines.

2. Please state what role the funders took in the study. If the funders had no role, please state: "The funders had no role in study design, data collection and analysis, decision to publish, or preparation of the manuscript." If this statement is not correct you must amend it as needed. Please include this amended Role of Funder statement in your cover letter; we will change the online submission form on your behalf.

*Response:*

Please change the submission form by adding the following statement regarding the funders' role: "The funders had no role in study design, data collection and analysis, decision to publish, or preparation of the manuscript."

3. If the reviewer comments include a recommendation to cite specific previously published works, please review and evaluate these publications to determine whether they are relevant and should be cited. There is no requirement to cite these works unless the editor has indicated otherwise.

*Response:*

There were no recommendations to cite additional publications.

**Editor Comments:**

First, while the combined SES–microbiome models reach statistical significance, the explained variance in cognitive outcomes remains modest (approximately 10%). The manuscript should more carefully contextualize these effect sizes and avoid language that may overstate their practical or developmental significance.

*Response:*

In accordance with the editor's suggestion, we have carefully revised the manuscript to avoid language that overstates the impact of SES and the microbiome on cognitive development, given the modest effect sizes derived from the models.

Second, the absence of key confounding variables, particularly dietary information and oral health status, substantially limits interpretation of the salivary microbiome findings. Given their known influence on oral microbial composition, this limitation should be more prominently acknowledged and critically discussed, especially in relation to the biological interpretations proposed.

*Response:*

In accordance with the editor's suggestion, we have acknowledged the importance of incorporating data on dietary patterns and oral health into the study. We have added a paragraph to the limitation section to read as follows:

" First, among the SES indicators, we lacked information on the participants' dietary exposures and oral health—factors that are closely intertwined and often conceptualized as a bidirectional cycle. Poor oral health can limit dietary choices, while inadequate nutrition can exacerbate oral diseases. Both play essential roles in shaping the microbiome and developmental processes and may substantially influence a child's cognitive function. Incorporating dietary patterns and oral health into further investigations of the SES-microbiome-cognition relationship is critical, as these factors act as primary mediators and modifiable risk factors."

Third, the restriction of the sample to Hispanic and African American participants, although methodologically motivated, has implications for generalizability and potential selection bias. This design decision requires clearer justification in the Methods and more explicit discussion as a limitation.

*Response:*

In the revised manuscript, we have addressed the editor's comment regarding the generalizability of the findings due to the sample's restrictions. We have added a limitation to the Discussion to read as follows:

" Third, the study sample is restricted to two ethnic groups—Hispanic and Black Americans—and represents a geographically local population from the greater NYC area. This restriction may affect the generalizability of our findings, particularly regarding associations between specific microbial taxa and their communities, as such relationships may vary across regions."

Finally, parts of the Discussion advance speculative mechanistic or interventional implications (e.g., oral–gut–brain pathways, cognitive health interventions) that are not directly supported by the cross-sectional data. These sections should be more cautiously framed and clearly distinguished from empirically supported conclusions.

*Response:*

We thank the editor for this comment. We have carefully revised the manuscript to ensure that the discussion of empirically supported results is distinguished from speculations on potential interventional implications. Also, we have removed the following sentence from the Abstract:

"In turn, addressing SES-related disparities, including those that challenge the formation of a healthy microbiome, could lead to improved cognitive health outcomes".

**Reviewers' comments:**

**Reviewer #1:**

1. There is no data availability statement in the manuscript. The raw metagenomic reads should be deposited in a public repository such as NCBI.

*Response:*

In accordance with the reviewer's suggestion, we have added a data availability statement to the revised version of the manuscript as follows: "The raw data of the microbiome sequencing reads have been deposited in the NCBI Sequence Read Archive (SRA) under the accession number PRJNA1428291."

2. Details are needed regarding the microbiome analyses, including how saliva samples were collected, as well as the DNA extraction, sequencing, and analysis methods.

*Response:*

In accordance with the reviewer's suggestion, we have provided details of the microbiome analyses. In the Methods section of the revised manuscript, we described the procedures for saliva sampling: spitting into the OG-500 Oragene-DNA collection kit, and DNA isolation: the Oragene/prepIT•L2P method as per the manufacturer's protocol. We specified the sequencing library preparation method: the Illumina TruSeq DNA PCR-Free, and the sequencing platform: Illumina NovaSeq 6000. Also, we provided details on the microbiome taxonomic profiling, including the MetaPhlAn tool version, the parameters used, and the reference database name: (vOct22 database).

3. Since the authors have metagenomic sequencing data, it would be beneficial for them to also include predicted functional analyses.

*Response:*

We thank the reviewer for his thoughtful suggestion to include predicted functional analyses. Having sequencing data, we could reconstruct the potential metabolic capacity of the microbiome and include metabolic pathways as an additional level of analysis, running separate models. However, this analysis would force us to speculate on the mechanisms underlying the associations between the microbiome and cognitive functioning. We believe this analysis would significantly shift the focus of the current manuscript. However, it has strong potential as a further standalone study, and we thank the reviewer for the insightful suggestion.

*Response:*

4. The beta-diversity can be used to reveal the oral microbiome in different socioeconomic status.

*Response:*

We thank the reviewer for this comment. We agree that the analysis of microbiome-SES associations can be performed using beta-diversity measures per se. In our analysis, beta-diversity, as a measure of interindividual similarity/dissimilarity, was assessed using co-occurrence networks and bacterial community distributions.

Minor comments:

1. In line 216, 'and their co-existing core Communities.' It should be 'communities'.

2. In line 300, 'The HBN data collection procedures are described in detail elsewhere.' It should be 'details'.

*Response:*

We have addressed these comments and made changes in the revised version of the manuscript.

**Reviewer #2:** I recommend that the manuscript adopt a more accessible and internationally oriented writing style to improve clarity for non-native English readers and students.

*Response:*

In accordance with the reviewer's suggestion, we have made numerous small changes throughout the manuscript, such as shortening sentences, replacing complex words, and ensuring logical paragraph transitions, which we hope will improve the manuscript's clarity and readability.

Additionally, the inclusion of more dynamic figures, tables, and analytical charts would strengthen the presentation of the results. Where possible, the figures should be updated to reflect the most recent analyses and data.

*Response:*

In accordance with the reviewer's suggestion, we have reviewed the figures and tables to ensure they present the most recent analysis and data reported in the paper.
