# Supplementary material for: Salivary microbiome, socioeconomic status, and cognitive performance: Triangulating the relationship
Source: PLoS One. 2026 Nov 2;21(11):e0361602. doi: 10.1371/journal.pone.0361602 (PMC13726965; doi:10.1371/journal.pone.0361602)
Supplement: Supplementary file 2 [file pone.0361602.s004.docx]

*Dear Editors and Reviewers,*

*We are grateful for your consideration of our manuscript entitled "Salivary microbiome, socioeconomic status, and cognitive performance: Triangulating the relationship." We appreciate the constructive comments on our paper. We have carefully studied each comment and made the corresponding revisions, which we hope will meet with your approval. Below, we provide our point-by-point responses to your comments.*

*Sincerely,*

*On behalf of the authors*

*Oxana Yu. Naumova, PhD*

**Editor Comments:**
Based on the reviewers' reports, I recommend major revision. Both reviewers identify issues that materially affect the reliability and interpretability of the manuscript. The taxonomic reporting requires substantial correction, as several taxa appear to be mislabeled or assigned to incorrect ranks, and the manuscript should be updated according to current bacterial nomenclature. In addition, the statistical presentation is incomplete, particularly where non-significant group differences are described without the corresponding analytical results. The authors must also ensure that the sequence data accession is publicly accessible. Finally, the second Reviewer raises a broader concern regarding the limited resolution and strength of the association analysis, particularly in the absence of species-level or functional analysis and given the apparently limited sample size. These points should be addressed before the manuscript can be reconsidered.

*Response:*

In accordance with the Editor's suggestions, we have thoroughly revised the manuscript and systematically addressed all comments and concerns. We have reviewed the text to ensure strict compliance with current bacterial nomenclature and to verify that all taxa are accurately labeled. In response to Reviewer3's request, we have added statistical details for non-significant inter-group comparisons. We have provided a temporary reviewer access key for our sequencing data repository deposited in the NCBI SRA under accession # PRJNA1428291: <https://dataview.ncbi.nlm.nih.gov/object/PRJNA1428291?reviewer=pqclijmam55ja95kjlfbe5ssi8>

We have addressed Reviewer1's broader concerns regarding our association analyses and the potential integration of public datasets, as follows. We performed a supplementary analysis investigating the association between metabolic pathway abundances (derived using HUMAnN 3.0) and cognitive phenotypes, incorporating these new findings into the Results section and Supplementary Table material. In our accompanying point-by-point response, we provide detailed justifications for our study design. Specifically, we clarify that: (1) association analyses were intentionally conducted at the genus level to minimize data sparsity and reduce dimensionality given our moderate sample size; and (2) our study was restricted to our specific cohort due to a lack of publicly available datasets providing the necessary individual-level intersection of all three primary variables (oral microbiome, SES, and normative cognitive functioning.

**Reviewers' comments:**

**Reviewer #1:**

1. The accession number PRJNA1428291 is not publicly available.

*Response:*

We apologize for the inconvenience; the dataset has been fully uploaded, but is currently under embargo until the manuscript is accepted for publication. To allow for peer review, we have generated a temporary reviewer access key. The data can be privately reviewed using the following link: <https://dataview.ncbi.nlm.nih.gov/object/PRJNA1428291?reviewer=pqclijmam55ja95kjlfbe5ssi8>

2. The authors did not accept my suggestion to use metagenomic data to perform functional analysis and to identify microbial taxa at the species level, which limits the significance of the work. If the study is primarily an association analysis at the genus level, the sample size is insufficient to support the conclusions. It should be feasible for the authors to include additional samples from public databases.

*Response:*

In accordance with the Reviewer's suggestion, functional profiling of metagenomic data via HUMAnN 3.0 was added to the manuscript, with metabolic pathway abundances detailed in Supplementary Table S9 and associations with cognitive phenotypes presented in the "Results: Microbiome and cognitive functioning" section. We reported a number of weak-to-moderate correlations between cognitive function scores and pathways related to amino acid, cofactor, and lipid biosynthesis and metabolism. In the Discussion section, we highlighted that our findings on the associations between cognitive function and L-arginine and S-adenosylmethionine (SAM) align with the literature.

Regarding the taxonomic level of the microbiome, we intentionally focused our association analysis at the genus level for the following critical methodological reasons. (1) Genus-level classification provides the most reliable and conservative taxonomic assignments, minimizing the alignment artifacts and misclassifications that frequently occur at the species level. (2) Microbial abundance data at the species level in our dataset exhibited a high level of severe zero-inflation. (3) Aggregating data at the genus level is a robust approach to reducing dimensionality in the microbiome. We think that, altogether, this helped us reduce the number of tests and increase our power to detect significant signals in the study with a moderate sample size.

We appreciate the Reviewer's suggestion to include supplementary samples from public databases to enhance statistical power. We agree that increasing sample sizes is a vital goal in microbiome and neurodevelopmental research. However, to our knowledge, there is a notable scarcity of multifaceted datasets that concurrently capture individual-level information on the oral microbiome, SES, and normative cognitive functioning. Because our study aimed to investigate how SES and the microbiome act as complementary contributors (and potentially interact) to shape cognitive outcomes, using disjointed datasets from different cohorts would introduce unmanageable confounding variables and severely limit causal inference. Consequently, we are restricted to our current cohort, which provides the necessary high-fidelity, individual-level intersection of all three variables.

**Reviewer #3:**

1. The authors inaccurately used the names of the obtained taxa and their taxonomic rank designations when describing their results. It is necessary to clarify the original name determined by systematic analysis and use this name. For example, L. 101: Saccharibacteria is an obsolete bacterial phylum name; its use in listing bacteria identified at the genus level is incorrect. Its correct name should be clarified based on the primary data. Similar comments apply to the data presented in Tables 1 and 2, where the following taxa are listed as bacterial genera: Candidatus Saccharibacteria, Ca. Nanosynsacchari, Ca. Saccharibacteria and Bacteroidetes are phyla, not genera; Clostridiales Fam XIII is an order; Bacteroidaceae is a family; and GGB1843 indicates a taxon. Similar comments: L. 167, 242-243. In addition, the captions to Figures 1 and 2 and the names of the taxa in the figures should be revised.

2. The authors used an old taxonomy of bacteria in the text of the manuscript (Saccharibacteria, Nanosynsacchari, Bacteroidetes); therefore, it is necessary to carefully proofread and make corrections in accordance with the current taxonomy. See https://www.bacterio.net/ and Oren A, Garrity GM. Valid publication of the names of forty-two phyla of prokaryotes. Int J Syst Evol Microbiol 2021; 71:5056.

*Response:*

We are grateful to the Reviewer for pointing out these inaccuracies in our taxonomic labeling and for directing us to the current prokaryotic nomenclature guidelines. We agree that higher-level taxonomic ranks were incorrectly formatted as genera, and that legacy phylum names were used. We have thoroughly reviewed the manuscript, figures, and tables to correct the taxonomic designations. Because our genus-level association analysis retained reads that could not be resolved down to a specific named genus, we have explicitly reformatted these entries to indicate their true taxonomic rank as "Unclassified" (Genus), precisely as they were originally assigned in the MetaPhlAn output. Specifically, the following corrections have been made:

The phylum name *Bacteroidetes* has been updated to the validly published phylum name *Bacteroidota*. In instances where it was listed as a genus, it has been corrected to *Bacteroidota* unclassified (or *Bacteroides*, where primary data supported it). *Candidatus Saccharibacteria* has been corrected to *Candidatus Saccharibacteria unclassified* and *Bacteroidaceae* – to *Bacteroidaceae unclassified* to clarify that it represents an unresolved genus-level lineage within that phylum or family. *Clostridiales Fam XIII* has been corrected to *Clostridiales Fam XIII Incertae Sedis unclassified*. *Ca. Nanosynsacchari* has been corrected to its proper genus designation, *Candidatus Nanosynsaccharibacterium*. To address the Reviewer's note regarding GGB1843, we have added a clarifying footnote to Table 1: "GGB1843 represents a Genus-level Genome Bin (GGB) defined within the MetaPhlAn profiling database as a distinct genus-level lineage within the family Bacteroidaceae."

3. L. 331, 348, 362: The authors state that no significant differences were found between the groups and provide a summary of descriptive statistics; however, Table 4 does not present the statistical analysis data. The statistical analysis results should be included to visualize all the differences.

*Response:*

In compliance with the Reviewer's recommendation, we have provided statistical values for non-significant results, wherever inter-group comparisons were reported.The updated section within the Methods:Sample now reads as follows: "Descriptive statistics for demographics across the four participant groups defined by sex and ethnicity are provided in Table 4. There were no significant differences in age distribution across sex and ethnic groups (*𝜒*^2^(3) = 3.219, *p* = 0.359) and in the sex ratio between the ethnic groups (*𝜒*^2^ = 1.306, *p* = 0.253)."

Within the Methods:Cognitive Assessments section now reads as follows: "Individual scores are presented in S8 Table; descriptive statistics for WISC scores across the four sex/ethnic participant groups are shown in Table 4. No significant group differences were revealed with the Kruskal-Wallis tests (df = 3; N = 200): *H*_FRI_ = 2.39, *p* = 0.495; *H*_PSI_ = 2.02, *p* = 0.568; *H*_VCI_ = 0.641, *p* = 0.887; *H*_VSI_ = 3.90, *p* = 0.272; *H*_WMI_ = 2.79, *p* = 0.425; *H*_NVIQ_ = 0.88, *p* = 0.830; *H*_FSIQ_ = 0.724, *p* = 0.868)."
