# Supplementary material for: Salivary microbiome, socioeconomic status, and cognitive performance: Triangulating the relationship
Source: PLoS One. 2026 Nov 2;21(11):e0361602. doi: 10.1371/journal.pone.0361602 (PMC13726965; doi:10.1371/journal.pone.0361602)
Supplement: Supplementary file 3 [file pone.0361602.s005.docx]

*Dear Editors and Reviewers,*

*We are grateful for your consideration of our manuscript entitled "Salivary microbiome, socioeconomic status, and cognitive performance: Triangulating the relationship." We have revised the manuscript and addressed the final points raised during the review process. Below, we provide our response to the Editor's comment and detail the corresponding revisions made to the text.*

*Sincerely,*

*On behalf of the authors*

*Oxana Yu. Naumova, PhD*

**Editor Comments:**

Having carefully reviewed the revised manuscript and the authors' point-by-point response, I am satisfied that the majority of the concerns raised in the previous round have been adequately addressed. The taxonomic nomenclature has been corrected throughout the text, tables, and figures in accordance with current bacterial taxonomy; statistical results for non-significant group comparisons have been added; the sequence data accession is appropriately reported in the Data Availability Statement; and a supplementary functional analysis (HUMAnN 3.0) has been incorporated to partially address the concern regarding analytical resolution.

However, while the authors provided a sound methodological justification in their response letter for restricting the association analysis to the genus level and for not incorporating additional public datasets, this justification has not been reflected in the manuscript itself. I therefore ask the authors to add a brief statement to the Limitations section acknowledging the genus-level resolution of the analysis and the potential constraints this, together with the sample size, places on statistical power and the generalizability of the findings. Pending this minor addition, the manuscript will be suitable for acceptance.

***Response:***

In accordance with the Editor's recommendation, we have added a dedicated statement to the Limitations section of the manuscript to explicitly acknowledge the genus-level resolution and the rationale for not merging our cohort with external public datasets, as well as the resulting constraints on statistical power and generalizability. The integrated paragraph now reads as follows (new text marked in bold):

Several limitations of this study should be noted. First, our SES indicators did not include dietary exposures and oral health—two closely intertwined factors that shape the microbiome through a bidirectional cycle. Poor oral health can restrict dietary choices, while inadequate nutrition can exacerbate oral diseases; both may substantially affect microbiome composition and developmental processes, thereby influencing a child's cognitive function. Future work should incorporate these variables as key mediators and modifiable risk factors. Second, the cross-sectional design precludes conclusions about causality or developmental trajectories. Longitudinal studies are needed to clarify the directionality and persistence of associations between the microbiome and cognitive performance. **Third, our taxonomic profiling and association analyses were intentionally restricted to the genus level. While this approach was adopted as a conservative strategy to ensure robust classification, mitigate severe zero-inflation, and reduce dimensionality to maintain statistical power, it inherently limits the taxonomic resolution of our findings regarding specific microbial species. Fourth, while a larger sample size obtained by incorporating public datasets would be highly desirable to maximize statistical power, the moderate size of our current cohort was a necessary trade-off. The unique and multi-faceted nature of our dataset—which concurrently captures individual-level data on the oral microbiome, SES, and normative cognitive functioning—made the integration of external data unfeasible without introducing unmanageable confounding factors.** Fifth, the sample was limited to Hispanic and Black American children from the greater NYC area, which may constrain the generalizability of the findings, particularly regarding associations between cognitive performance and specific microbial taxa and their communities, as such relationships may vary across regions. Finally, interpretation is limited by the relatively small body of existing research on the interplay among the oral microbiome, SES, and cognition.

**Reviewers' comments:** No further comments.
