# Supplementary material for: Salivary microbiome, socioeconomic status, and cognitive performance: Triangulating the relationship
Source: PLoS One. 2026 Nov 2;21(11):e0361602. doi: 10.1371/journal.pone.0361602 (PMC13726965; doi:10.1371/journal.pone.0361602)
Supplement: Peer review history

# Peer review: Decision Letter 0

**DOI:** 10.1371/journal.pone.0361602.r001

**Authors:** Antonio Magan-Fernandez

4 Feb 2026

Dear Dr. Naumova,

Thank you for submitting your manuscript to PLOS ONE. After careful consideration, we feel that it has merit but does not fully meet PLOS ONE’s publication criteria as it currently stands. Therefore, we invite you to submit a revised version of the manuscript that addresses the points raised during the review process.

Please submit your revised manuscript by Mar 21 2026 11:59PM. If you will need more time than this to complete your revisions, please reply to this message or contact the journal office at plosone@plos.org. When you're ready to submit your revision, log on to https://www.editorialmanager.com/pone/ and select the 'Submissions Needing Revision' folder to locate your manuscript file.

- A letter that responds to each point raised by the academic editor and reviewer(s). You should upload this letter as a separate file labeled 'Response to Reviewers'.
- A marked-up copy of your manuscript that highlights changes made to the original version. You should upload this as a separate file labeled 'Revised Manuscript with Track Changes'.
- An unmarked version of your revised paper without tracked changes. You should upload this as a separate file labeled 'Manuscript'.

If you would like to make changes to your financial disclosure, please include your updated statement in your cover letter. Guidelines for resubmitting your figure files are available below the reviewer comments at the end of this letter.

If applicable, we recommend that you deposit your laboratory protocols in protocols.io to enhance the reproducibility of your results. Protocols.io assigns your protocol its own identifier (DOI) so that it can be cited independently in the future. For instructions see: https://journals.plos.org/plosone/s/submission-guidelines#loc-laboratory-protocols. Additionally, PLOS ONE offers an option for publishing peer-reviewed Lab Protocol articles, which describe protocols hosted on protocols.io. Read more information on sharing protocols at https://plos.org/protocols?utm_medium=editorial-email&utm_source=authorletters&utm_campaign=protocols.

We look forward to receiving your revised manuscript.

Kind regards,

Antonio Magan-Fernandez, DDS, MSc, PhD

Academic Editor

PLOS One

Journal Requirements:

When submitting your revision, we need you to address these additional requirements.

1. Please ensure that your manuscript meets PLOS ONE's style requirements, including those for file naming. The PLOS ONE style templates can be found at

https://journals.plos.org/plosone/s/file?id=wjVg/PLOSOne_formatting_sample_main_body.pdf and

https://journals.plos.org/plosone/s/file?id=ba62/PLOSOne_formatting_sample_title_authors_affiliations.pdf

2. Thank you for stating the following financial disclosure:

This study was supported by grants from the Eunice Kennedy Shriver National Institute of Child Health and Human Development (NICHD) to the University of Houston and the Texas Center for Learning Disabilities (P50HD052117 to Jack M. Fletcher), Florida State University (P50HD052120 to Richard Wagner), and Baylor College of Medicine (P20HD091005 to Elena L. Grigorenko).

Please state what role the funders took in the study. If the funders had no role, please state: "The funders had no role in study design, data collection and analysis, decision to publish, or preparation of the manuscript."

If this statement is not correct you must amend it as needed.

Please include this amended Role of Funder statement in your cover letter; we will change the online submission form on your behalf.

3. If the reviewer comments include a recommendation to cite specific previously published works, please review and evaluate these publications to determine whether they are relevant and should be cited. There is no requirement to cite these works unless the editor has indicated otherwise.

Additional Editor Comments:

After evaluation of the manuscript “Salivary microbiome, socioeconomic status, and cognitive performance: Triangulating the relationship”, I find that the study addresses a relevant and interdisciplinary research question and meets PLOS ONE’s criteria for technical soundness. The use of a well-characterized public cohort, appropriate metagenomic methods, and transparent statistical analyses supports the overall validity of the work

However, before the manuscript can be considered further, several substantive issues related to interpretation, framing, and study limitations must be addressed.

First, while the combined SES–microbiome models reach statistical significance, the explained variance in cognitive outcomes remains modest (approximately 10%). The manuscript should more carefully contextualize these effect sizes and avoid language that may overstate their practical or developmental significance.

Second, the absence of key confounding variables, particularly dietary information and oral health status, substantially limits interpretation of the salivary microbiome findings. Given their known influence on oral microbial composition, this limitation should be more prominently acknowledged and critically discussed, especially in relation to the biological interpretations proposed.

Third, the restriction of the sample to Hispanic and African American participants, although methodologically motivated, has implications for generalizability and potential selection bias. This design decision requires clearer justification in the Methods and more explicit discussion as a limitation.

Finally, parts of the Discussion advance speculative mechanistic or interventional implications (e.g., oral–gut–brain pathways, cognitive health interventions) that are not directly supported by the cross-sectional data. These sections should be more cautiously framed and clearly distinguished from empirically supported conclusions.

I therefore recommend Major Revision and invite the authors to submit a revised manuscript that adequately addresses these concerns.

[Note: HTML markup is below. Please do not edit.]

Reviewers' comments:

Reviewer's Responses to Questions

**Comments to the Author**

1. Is the manuscript technically sound, and do the data support the conclusions?

Reviewer #1: Partly

Reviewer #2: Yes

**********

2. Has the statistical analysis been performed appropriately and rigorously?

Reviewer #1: Yes

Reviewer #2: I Don't Know

**********

3. Have the authors made all data underlying the findings in their manuscript fully available?

The PLOS Data policy

Reviewer #1: No

Reviewer #2: No

**********

4. Is the manuscript presented in an intelligible fashion and written in standard English?

Reviewer #1: Yes

Reviewer #2: Yes

**********

Reviewer #1: The manuscript titled “Salivary Microbiome, Socioeconomic Status, and Cognitive Performance: Triangulating the Relationship” investigates the association between children’s oral microbiome and cognitive performance. The writing is generally clear, and the analyses are appropriately conducted. However, several areas still require improvement.

Major comments:

1. There is no data availability statement in the manuscript. The raw metagenomic reads should be deposited in a public repository such as NCBI.

2. Details are needed regarding the microbiome analyses, including how saliva samples were collected, as well as the DNA extraction, sequencing, and analysis methods.

3. Since the authors have metagenomic sequencing data, it would be beneficial for them to also include predicted functional analyses.

4. The beta-diversity can be used to reveal the oral microbiome in different socioeconomic status.

Minor comments

1. In line 216, ‘and their co-existing core Communities.’ It should be ‘communities’.

2. In line 300, ‘The HBN data collection procedures are described in detail elsewhere.’ It should be ‘details’.

Reviewer #2: I recommend that the manuscript adopt a more accessible and internationally oriented writing style to improve clarity for non-native English readers and students.

Additionally, the inclusion of more dynamic figures, tables, and analytical charts would strengthen the presentation of the results. Where possible, the figures should be updated to reflect the most recent analyses and data.

**********

what does this mean?). If published, this will include your full peer review and any attached files.

If you choose “no”, your identity will remain anonymous but your review may still be made public.

**Do you want your identity to be public for this peer review?**  For information about this choice, including consent withdrawal, please see our Privacy Policy

Reviewer #1: No

Reviewer #2: No

**********

[NOTE: If reviewer comments were submitted as an attachment file, they will be attached to this email and accessible via the submission site. Please log into your account, locate the manuscript record, and check for the action link "View Attachments". If this link does not appear, there are no attachment files.]

To ensure your figures meet our technical requirements, please review our figure guidelines: https://journals.plos.org/plosone/s/figures

You may also use PLOS’s free figure tool, NAAS, to help you prepare publication quality figures: https://journals.plos.org/plosone/s/figures#loc-tools-for-figure-preparation.

NAAS will assess whether your figures meet our technical requirements by comparing each figure against our figure specifications.

# Author comment: Author response to Decision Letter 1

**DOI:** 10.1371/journal.pone.0361602.r002

23 Mar 2026

Journal Requirements:

1. Please ensure that your manuscript meets PLOS ONE's style requirements, including those for file naming.

Response:

We have revised and formatted the manuscript in accordance with PLOS ONE's formatting guidelines.

2. Please state what role the funders took in the study. If the funders had no role, please state: "The funders had no role in study design, data collection and analysis, decision to publish, or preparation of the manuscript." If this statement is not correct you must amend it as needed. Please include this amended Role of Funder statement in your cover letter; we will change the online submission form on your behalf.

Response:

Please change the submission form by adding the following statement regarding the funders' role: "The funders had no role in study design, data collection and analysis, decision to publish, or preparation of the manuscript."

3. If the reviewer comments include a recommendation to cite specific previously published works, please review and evaluate these publications to determine whether they are relevant and should be cited. There is no requirement to cite these works unless the editor has indicated otherwise.

Response:

There were no recommendations to cite additional publications.

Editor Comments:

First, while the combined SES–microbiome models reach statistical significance, the explained variance in cognitive outcomes remains modest (approximately 10%). The manuscript should more carefully contextualize these effect sizes and avoid language that may overstate their practical or developmental significance.

Response:

In accordance with the editor's suggestion, we have carefully revised the manuscript to avoid language that overstates the impact of SES and the microbiome on cognitive development, given the modest effect sizes derived from the models.

Second, the absence of key confounding variables, particularly dietary information and oral health status, substantially limits interpretation of the salivary microbiome findings. Given their known influence on oral microbial composition, this limitation should be more prominently acknowledged and critically discussed, especially in relation to the biological interpretations proposed.

Response:

In accordance with the editor's suggestion, we have acknowledged the importance of incorporating data on dietary patterns and oral health into the study. We have added a paragraph to the limitation section to read as follows:

" First, among the SES indicators, we lacked information on the participants' dietary exposures and oral health—factors that are closely intertwined and often conceptualized as a bidirectional cycle. Poor oral health can limit dietary choices, while inadequate nutrition can exacerbate oral diseases. Both play essential roles in shaping the microbiome and developmental processes and may substantially influence a child's cognitive function. Incorporating dietary patterns and oral health into further investigations of the SES-microbiome-cognition relationship is critical, as these factors act as primary mediators and modifiable risk factors."

Third, the restriction of the sample to Hispanic and African American participants, although methodologically motivated, has implications for generalizability and potential selection bias. This design decision requires clearer justification in the Methods and more explicit discussion as a limitation.

Response:

In the revised manuscript, we have addressed the editor's comment regarding the generalizability of the findings due to the sample's restrictions. We have added a limitation to the Discussion to read as follows:

" Third, the study sample is restricted to two ethnic groups—Hispanic and Black Americans—and represents a geographically local population from the greater NYC area. This restriction may affect the generalizability of our findings, particularly regarding associations between specific microbial taxa and their communities, as such relationships may vary across regions."

Finally, parts of the Discussion advance speculative mechanistic or interventional implications (e.g., oral–gut–brain pathways, cognitive health interventions) that are not directly supported by the cross-sectional data. These sections should be more cautiously framed and clearly distinguished from empirically supported conclusions.

Response:

We thank the editor for this comment. We have carefully revised the manuscript to ensure that the discussion of empirically supported results is distinguished from speculations on potential interventional implications. Also, we have removed the following sentence from the Abstract:

"In turn, addressing SES-related disparities, including those that challenge the formation of a healthy microbiome, could lead to improved cognitive health outcomes".

Reviewers' comments:

Reviewer #1:

1. There is no data availability statement in the manuscript. The raw metagenomic reads should be deposited in a public repository such as NCBI.

Response:

In accordance with the reviewer's suggestion, we have added a data availability statement to the revised version of the manuscript as follows: "The raw data of the microbiome sequencing reads have been deposited in the NCBI Sequence Read Archive (SRA) under the accession number PRJNA1428291."

2. Details are needed regarding the microbiome analyses, including how saliva samples were collected, as well as the DNA extraction, sequencing, and analysis methods.

Response:

In accordance with the reviewer's suggestion, we have provided details of the microbiome analyses. In the Methods section of the revised manuscript, we described the procedures for saliva sampling: spitting into the OG-500 Oragene-DNA collection kit, and DNA isolation: the Oragene/prepIT•L2P method as per the manufacturer's protocol. We specified the sequencing library preparation method: the Illumina TruSeq DNA PCR-Free, and the sequencing platform: Illumina NovaSeq 6000. Also, we provided details on the microbiome taxonomic profiling, including the MetaPhlAn tool version, the parameters used, and the reference database name: (vOct22 database).

3. Since the authors have metagenomic sequencing data, it would be beneficial for them to also include predicted functional analyses.

Response:

We thank the reviewer for his thoughtful suggestion to include predicted functional analyses. Having sequencing data, we could reconstruct the potential metabolic capacity of the microbiome and include metabolic pathways as an additional level of analysis, running separate models. However, this analysis would force us to speculate on the mechanisms underlying the associations between the microbiome and cognitive functioning. We believe this analysis would significantly shift the focus of the current manuscript. However, it has strong potential as a further standalone study, and we thank the reviewer for the insightful suggestion.

4. The beta-diversity can be used to reveal the oral microbiome in different socioeconomic status.

Response:

We thank the reviewer for this comment. We agree that the analysis of microbiome-SES associations can be performed using beta-diversity measures per se. In our analysis, beta-diversity, as a measure of interindividual similarity/dissimilarity, was assessed using co-occurrence networks and bacterial community distributions.

Minor comments:

1. In line 216, 'and their co-existing core Communities.' It should be 'communities'.

2. In line 300, 'The HBN data collection procedures are described in detail elsewhere.' It should be 'details'.

Response:

We have addressed these comments and made changes in the revised version of the manuscript.

Reviewer #2:

I recommend that the manuscript adopt a more accessible and internationally oriented writing style to improve clarity for non-native English readers and students.

Response:

In accordance with the reviewer's suggestion, we have made numerous small changes throughout the manuscript, such as shortening sentences, replacing complex words, and ensuring logical paragraph transitions, which we hope will improve the manuscript's clarity and readability.

Additionally, the inclusion of more dynamic figures, tables, and analytical charts would strengthen the presentation of the results. Where possible, the figures should be updated to reflect the most recent analyses and data.

Response:

In accordance with the reviewer's suggestion, we have reviewed the figures and tables to ensure they present the most recent analysis and data reported in the paper.

Attachment Submitted filename: Response to Reviewers_PONE-D-25-36917.docx

# Peer review: Decision Letter 1

**DOI:** 10.1371/journal.pone.0361602.r003

**Authors:** Antonio Magan-Fernandez

6 May 2026

Dear Dr. Naumova,

Thank you for submitting your manuscript to PLOS ONE. After careful consideration, we feel that it has merit but does not fully meet PLOS ONE’s publication criteria as it currently stands. Therefore, we invite you to submit a revised version of the manuscript that addresses the points raised during the review process.

Please submit your revised manuscript by Jun 20 2026 11:59PM. If you will need more time than this to complete your revisions, please reply to this message or contact the journal office at plosone@plos.org. When you're ready to submit your revision, log on to https://www.editorialmanager.com/pone/ and select the 'Submissions Needing Revision' folder to locate your manuscript file.

- A letter that responds to each point raised by the academic editor and reviewer(s). You should upload this letter as a separate file labeled 'Response to Reviewers'.
- A marked-up copy of your manuscript that highlights changes made to the original version. You should upload this as a separate file labeled 'Revised Manuscript with Track Changes'.
- An unmarked version of your revised paper without tracked changes. You should upload this as a separate file labeled 'Manuscript'.

If you would like to make changes to your financial disclosure, please include your updated statement in your cover letter. Guidelines for resubmitting your figure files are available below the reviewer comments at the end of this letter.

If applicable, we recommend that you deposit your laboratory protocols in protocols.io to enhance the reproducibility of your results. Protocols.io assigns your protocol its own identifier (DOI) so that it can be cited independently in the future. For instructions see: https://journals.plos.org/plosone/s/submission-guidelines#loc-laboratory-protocols. Additionally, PLOS ONE offers an option for publishing peer-reviewed Lab Protocol articles, which describe protocols hosted on protocols.io. Read more information on sharing protocols at https://plos.org/protocols?utm_medium=editorial-email&utm_source=authorletters&utm_campaign=protocols.

As the corresponding author, your ORCID iD is verified in the submission system and will appear in the published article. PLOS supports the use of ORCID, and we encourage all coauthors to register for an ORCID iD and use it as well. Please encourage your coauthors to verify their ORCID iD within the submission system before final acceptance, as unverified ORCID iDs will not appear in the published article. *Only*  the individual author can complete the verification step; PLOS staff *cannot*  verify ORCID iDs on behalf of authors.

We look forward to receiving your revised manuscript.

Kind regards,

Antonio Magan-Fernandez, DDS, MSc, PhD

Academic Editor

PLOS One

Journal Requirements:

If the reviewer comments include a recommendation to cite specific previously published works, please review and evaluate these publications to determine whether they are relevant and should be cited. There is no requirement to cite these works unless the editor has indicated otherwise.

Additional Editor Comments (if provided):

Based on the reviewers’ reports, I recommend major revision. Both reviewers identify issues that materially affect the reliability and interpretability of the manuscript. The taxonomic reporting requires substantial correction, as several taxa appear to be mislabeled or assigned to incorrect ranks, and the manuscript should be updated according to current bacterial nomenclature. In addition, the statistical presentation is incomplete, particularly where non-significant group differences are described without the corresponding analytical results. The authors must also ensure that the sequence data accession is publicly accessible. Finally, the second reviewer raises a broader concern regarding the limited resolution and strength of the association analysis, particularly in the absence of species-level or functional analysis and given the apparently limited sample size. These points should be addressed before the manuscript can be reconsidered.

[Note: HTML markup is below. Please do not edit.]

Reviewers' comments:

Reviewer's Responses to Questions

**Comments to the Author**

Reviewer #1: (No Response)

Reviewer #3: (No Response)

**********

2. Is the manuscript technically sound, and do the data support the conclusions?

Reviewer #1: Partly

Reviewer #3: Yes

**********

3. Has the statistical analysis been performed appropriately and rigorously?

Reviewer #1: Yes

Reviewer #3: Yes

**********

4. Have the authors made all data underlying the findings in their manuscript fully available?

The PLOS Data policy

Reviewer #1: No

Reviewer #3: Yes

**********

5. Is the manuscript presented in an intelligible fashion and written in standard English?

Reviewer #1: Yes

Reviewer #3: Yes

**********

Reviewer #1: 1. The accession number PRJNA1428291 is not publicly available.

2. The authors did not accept my suggestion to use metagenomic data to perform functional analysis and to identify microbial taxa at the species level, which limits the significance of the work. If the study is primarily an association analysis at the genus level, the sample size is insufficient to support the conclusions. It should be feasible for the authors to include additional samples from public databases.

Reviewer #3: This peer-reviewed manuscript examined the associations between oral microbiome diversity, socioeconomic status, and cognitive impairment as assessed by the Wechsler Intelligence Scale for Children in Hispanic and African American children aged 6-16 years. In the introduction, the authors convincingly argue the relevance of this study. Despite its status as a less stable microbial biotope, the oral cavity is the least invasive biotope among those currently being studied, and a very large number of health-related indicators can be directly assessed in saliva, thus establishing more direct relationships between these indicators and microbial community diversity and functioning.

I have several technical comments.

1. The authors inaccurately used the names of the obtained taxa and their taxonomic rank designations when describing their results. It is necessary to clarify the original name determined by systematic analysis and use this name. For example, L. 101: Saccharibacteria is an obsolete bacterial phylum name; its use in listing bacteria identified at the genus level is incorrect. Its correct name should be clarified based on the primary data. Similar comments apply to the data presented in Tables 1 and 2, where the following taxa are listed as bacterial genera: Candidatus Saccharibacteria, Ca. Nanosynsacchari, Ca. Saccharibacteria and Bacteroidetes are phyla, not genera; Clostridiales Fam XIII is an order; Bacteroidaceae is a family; and GGB1843 indicates a taxon. Similar comments: L. 167, 242-243. In addition, the captions to Figures 1 and 2 and the names of the taxa in the figures should be revised.

2. The authors used an old taxonomy of bacteria in the text of the manuscript (Saccharibacteria, Nanosynsacchari, Bacteroidetes); therefore, it is necessary to carefully proofread and make corrections in accordance with the current taxonomy. See https://www.bacterio.net/ and Oren A, Garrity GM. Valid publication of the names of forty-two phyla of prokaryotes. Int J Syst Evol Microbiol 2021; 71:5056.

3. L. 331, 348, 362: The authors state that no significant differences were found between the groups and provide a summary of descriptive statistics; however, Table 4 does not present the statistical analysis data. The statistical analysis results should be included to visualize all the differences.

**********

what does this mean?). If published, this will include your full peer review and any attached files.

If you choose “no”, your identity will remain anonymous but your review may still be made public.

**Do you want your identity to be public for this peer review?**  For information about this choice, including consent withdrawal, please see our Privacy Policy

Reviewer #1: No

Reviewer #3: No

**********

[NOTE: If reviewer comments were submitted as an attachment file, they will be attached to this email and accessible via the submission site. Please log into your account, locate the manuscript record, and check for the action link "View Attachments". If this link does not appear, there are no attachment files.]

To ensure your figures meet our technical requirements, please review our figure guidelines: https://journals.plos.org/plosone/s/figures

You may also use PLOS’s free figure tool, NAAS, to help you prepare publication quality figures: https://journals.plos.org/plosone/s/figures#loc-tools-for-figure-preparation.

NAAS will assess whether your figures meet our technical requirements by comparing each figure against our figure specifications.

# Author comment: Author response to Decision Letter 2

**DOI:** 10.1371/journal.pone.0361602.r004

26 Jun 2026

Dear Editors and Reviewers,

We appreciate the constructive comments on our paper.

We have carefully studied each comment and made the corresponding revisions.

Please find below our point-by-point responses to your comments.

Editor Comments:

Based on the reviewers' reports, I recommend major revision. Both reviewers identify issues that materially affect the reliability and interpretability of the manuscript. The taxonomic reporting requires substantial correction, as several taxa appear to be mislabeled or assigned to incorrect ranks, and the manuscript should be updated according to current bacterial nomenclature. In addition, the statistical presentation is incomplete, particularly where non-significant group differences are described without the corresponding analytical results. The authors must also ensure that the sequence data accession is publicly accessible. Finally, the second Reviewer raises a broader concern regarding the limited resolution and strength of the association analysis, particularly in the absence of species-level or functional analysis and given the apparently limited sample size. These points should be addressed before the manuscript can be reconsidered.

Response:

In accordance with the Editor's suggestions, we have thoroughly revised the manuscript and systematically addressed all comments and concerns. We have reviewed the text to ensure strict compliance with current bacterial nomenclature and to verify that all taxa are accurately labeled. In response to Reviewer3's request, we have added statistical details for non-significant inter-group comparisons. We have provided a temporary reviewer access key for our sequencing data repository deposited in the NCBI SRA under accession # PRJNA1428291: https://dataview.ncbi.nlm.nih.gov/object/PRJNA1428291?reviewer=pqclijmam55ja95kjlfbe5ssi8

We have addressed Reviewer1's broader concerns regarding our association analyses and the potential integration of public datasets, as follows. We performed a supplementary analysis investigating the association between metabolic pathway abundances (derived using HUMAnN 3.0) and cognitive phenotypes, incorporating these new findings into the Results section and Supplementary Table material. In our accompanying point-by-point response, we provide detailed justifications for our study design. Specifically, we clarify that: (1) association analyses were intentionally conducted at the genus level to minimize data sparsity and reduce dimensionality given our moderate sample size; and (2) our study was restricted to our specific cohort due to a lack of publicly available datasets providing the necessary individual-level intersection of all three primary variables (oral microbiome, SES, and normative cognitive functioning.

Reviewers' comments:

Reviewer #1:

1. The accession number PRJNA1428291 is not publicly available.

Response:

We apologize for the inconvenience; the dataset has been fully uploaded, but is currently under embargo until the manuscript is accepted for publication. To allow for peer review, we have generated a temporary reviewer access key. The data can be privately reviewed using the following link: https://dataview.ncbi.nlm.nih.gov/object/PRJNA1428291?reviewer=pqclijmam55ja95kjlfbe5ssi8

2. The authors did not accept my suggestion to use metagenomic data to perform functional analysis and to identify microbial taxa at the species level, which limits the significance of the work. If the study is primarily an association analysis at the genus level, the sample size is insufficient to support the conclusions. It should be feasible for the authors to include additional samples from public databases.

Response:

In accordance with the Reviewer's suggestion, functional profiling of metagenomic data via HUMAnN 3.0 was added to the manuscript, with metabolic pathway abundances detailed in Supplementary Table S9 and associations with cognitive phenotypes presented in the "Results: Microbiome and cognitive functioning" section. We reported a number of weak-to-moderate correlations between cognitive function scores and pathways related to amino acid, cofactor, and lipid biosynthesis and metabolism. In the Discussion section, we highlighted that our findings on the associations between cognitive function and L-arginine and S-adenosylmethionine (SAM) align with the literature.

Regarding the taxonomic level of the microbiome, we intentionally focused our association analysis at the genus level for the following critical methodological reasons. (1) Genus-level classification provides the most reliable and conservative taxonomic assignments, minimizing the alignment artifacts and misclassifications that frequently occur at the species level. (2) Microbial abundance data at the species level in our dataset exhibited a high level of severe zero-inflation. (3) Aggregating data at the genus level is a robust approach to reducing dimensionality in the microbiome. We think that, altogether, this helped us reduce the number of tests and increase our power to detect significant signals in the study with a moderate sample size.

We appreciate the Reviewer's suggestion to include supplementary samples from public databases to enhance statistical power. We agree that increasing sample sizes is a vital goal in microbiome and neurodevelopmental research. However, to our knowledge, there is a notable scarcity of multifaceted datasets that concurrently capture individual-level information on the oral microbiome, SES, and normative cognitive functioning. Because our study aimed to investigate how SES and the microbiome act as complementary contributors (and potentially interact) to shape cognitive outcomes, using disjointed datasets from different cohorts would introduce unmanageable confounding variables and severely limit causal inference. Consequently, we are restricted to our current cohort, which provides the necessary high-fidelity, individual-level intersection of all three variables.

Reviewer #3:

1. The authors inaccurately used the names of the obtained taxa and their taxonomic rank designations when describing their results. It is necessary to clarify the original name determined by systematic analysis and use this name. For example, L. 101: Saccharibacteria is an obsolete bacterial phylum name; its use in listing bacteria identified at the genus level is incorrect. Its correct name should be clarified based on the primary data. Similar comments apply to the data presented in Tables 1 and 2, where the following taxa are listed as bacterial genera: Candidatus Saccharibacteria, Ca. Nanosynsacchari, Ca. Saccharibacteria and Bacteroidetes are phyla, not genera; Clostridiales Fam XIII is an order; Bacteroidaceae is a family; and GGB1843 indicates a taxon. Similar comments: L. 167, 242-243. In addition, the captions to Figures 1 and 2 and the names of the taxa in the figures should be revised.

2. The authors used an old taxonomy of bacteria in the text of the manuscript (Saccharibacteria, Nanosynsacchari, Bacteroidetes); therefore, it is necessary to carefully proofread and make corrections in accordance with the current taxonomy. See https://www.bacterio.net/ and Oren A, Garrity GM. Valid publication of the names of forty-two phyla of prokaryotes. Int J Syst Evol Microbiol 2021; 71:5056.

Response:

We are grateful to the Reviewer for pointing out these inaccuracies in our taxonomic labeling and for directing us to the current prokaryotic nomenclature guidelines. We agree that higher-level taxonomic ranks were incorrectly formatted as genera, and that legacy phylum names were used. We have thoroughly reviewed the manuscript, figures, and tables to correct the taxonomic designations. Because our genus-level association analysis retained reads that could not be resolved down to a specific named genus, we have explicitly reformatted these entries to indicate their true taxonomic rank as "Unclassified" (Genus), precisely as they were originally assigned in the MetaPhlAn output. Specifically, the following corrections have been made:

The phylum name Bacteroidetes has been updated to the validly published phylum name Bacteroidota. In instances where it was listed as a genus, it has been corrected to Bacteroidota unclassified (or Bacteroides, where primary data supported it). Candidatus Saccharibacteria has been corrected to Candidatus Saccharibacteria unclassified and Bacteroidaceae – to Bacteroidaceae unclassified to clarify that it represents an unresolved genus-level lineage within that phylum or family. Clostridiales Fam XIII has been corrected to Clostridiales Fam XIII Incertae Sedis unclassified. Ca. Nanosynsacchari has been corrected to its proper genus designation, Candidatus Nanosynsaccharibacterium. To address the Reviewer's note regarding GGB1843, we have added a clarifying footnote to Table 1: "GGB1843 represents a Genus-level Genome Bin (GGB) defined within the MetaPhlAn profiling database as a distinct genus-level lineage within the family Bacteroidaceae."

3. L. 331, 348, 362: The authors state that no significant differences were found between the groups and provide a summary of descriptive statistics; however, Table 4 does not present the statistical analysis data. The statistical analysis results should be included to visualize all the differences.

Response:

In compliance with the Reviewer's recommendation, we have provided statistical values for non-significant results, wherever inter-group comparisons were reported.The updated section within the Methods:Sample now reads as follows: "Descriptive statistics for demographics across the four participant groups defined by sex and ethnicity are provided in Table 4. There were no significant differences in age distribution across sex and ethnic groups (𝜒2(3) = 3.219, p = 0.359) and in the sex ratio between the ethnic groups (𝜒2 = 1.306, p = 0.253)."

Within the Methods:Cognitive Assessments section now reads as follows: "Individual scores are presented in S8 Table; descriptive statistics for WISC scores across the four sex/ethnic participant groups are shown in Table 4. No significant group differences were revealed with the Kruskal-Wallis tests (df = 3; N = 200): HFRI = 2.39, p = 0.495; HPSI = 2.02, p = 0.568; HVCI = 0.641, p = 0.887; HVSI = 3.90, p = 0.272; HWMI = 2.79, p = 0.425; HNVIQ = 0.88, p = 0.830; HFSIQ = 0.724, p = 0.868)."

Attachment Submitted filename: Response to Reviewers_PONE-D-25-36917_Revision2.docx

# Peer review: Decision Letter 2

**DOI:** 10.1371/journal.pone.0361602.r005

**Authors:** Antonio Magan-Fernandez

8 Sep 2026

Dear Dr. Naumova,

Thank you for submitting your manuscript to PLOS One. After careful consideration, we feel that it has merit but does not fully meet PLOS One’s publication criteria as it currently stands. Therefore, we invite you to submit a revised version of the manuscript that addresses the points raised during the review process.

Please submit your revised manuscript by Oct 23 2026 11:59PM. If you will need more time than this to complete your revisions, please reply to this message or contact the journal office at plosone@plos.org. When you're ready to submit your revision, log on to https://www.editorialmanager.com/pone/ and select the 'Submissions Needing Revision' folder to locate your manuscript file.

- A letter that responds to each point raised by the academic editor and reviewer(s). You should upload this letter as a separate file labeled 'Response to Reviewers'.
- A marked-up copy of your manuscript that highlights changes made to the original version. You should upload this as a separate file labeled 'Revised Manuscript with Track Changes'.
- An unmarked version of your revised paper without tracked changes. You should upload this as a separate file labeled 'Manuscript'.

If you would like to make changes to your financial disclosure, please include your updated statement in your cover letter. Guidelines for resubmitting your figure files are available below the reviewer comments at the end of this letter.

If applicable, we recommend that you deposit your laboratory protocols in protocols.io to enhance the reproducibility of your results. Protocols.io assigns your protocol its own identifier (DOI) so that it can be cited independently in the future. For instructions see: https://journals.plos.org/plosone/s/submission-guidelines#loc-laboratory-protocols. Additionally, PLOS One offers an option for publishing peer-reviewed Lab Protocol articles, which describe protocols hosted on protocols.io. Read more information on sharing protocols at https://plos.org/protocols?utm_medium=editorial-email&utm_source=authorletters&utm_campaign=protocols.

As the corresponding author, your ORCID iD is verified in the submission system and will appear in the published article. PLOS supports the use of ORCID, and we encourage all coauthors to register for an ORCID iD and use it as well. Please encourage your coauthors to verify their ORCID iD within the submission system before final acceptance, as unverified ORCID iDs will not appear in the published article. *Only*  the individual author can complete the verification step; PLOS staff *cannot*  verify ORCID iDs on behalf of authors.

We look forward to receiving your revised manuscript.

Kind regards,

Antonio Magan-Fernandez, DDS, MSc, PhD

Academic Editor

PLOS One

Journal Requirements:

1. If the reviewer comments include a recommendation to cite specific previously published works, please review and evaluate these publications to determine whether they are relevant and should be cited. There is no requirement to cite these works unless the editor has indicated otherwise.

2. Please review your reference list to ensure that it is complete and correct. If you have cited papers that have been retracted, please include the rationale for doing so in the manuscript text, or remove these references and replace them with relevant current references. Any changes to the reference list should be mentioned in the rebuttal letter that accompanies your revised manuscript. If you need to cite a retracted article, indicate the article’s retracted status in the References list and also include a citation and full reference for the retraction notice.

Additional Editor Comments:

Having carefully reviewed the revised manuscript and the authors' point-by-point response, I am satisfied that the majority of the concerns raised in the previous round have been adequately addressed. The taxonomic nomenclature has been corrected throughout the text, tables, and figures in accordance with current bacterial taxonomy; statistical results for non-significant group comparisons have been added; the sequence data accession is appropriately reported in the Data Availability Statement; and a supplementary functional analysis (HUMAnN 3.0) has been incorporated to partially address the concern regarding analytical resolution. However, while the authors provided a sound methodological justification in their response letter for restricting the association analysis to the genus level and for not incorporating additional public datasets, this justification has not been reflected in the manuscript itself. I therefore ask the authors to add a brief statement to the Limitations section acknowledging the genus-level resolution of the analysis and the potential constraints this, together with the sample size, places on statistical power and the generalizability of the findings. Pending this minor addition, the manuscript will be suitable for acceptance.

[Note: HTML markup is below. Please do not edit.]

Reviewers' comments:

Reviewer's Responses to Questions

**Comments to the Author**

Reviewer #3: All comments have been addressed

**********

2. Is the manuscript technically sound, and do the data support the conclusions?

Reviewer #3: (No Response)

**********

3. Has the statistical analysis been performed appropriately and rigorously?

Reviewer #3: (No Response)

**********

4. Have the authors made all data underlying the findings in their manuscript fully available?

The PLOS Data policy

Reviewer #3: (No Response)

**********

5. Is the manuscript presented in an intelligible fashion and written in standard English?

Reviewer #3: (No Response)

**********

Reviewer #3: (No Response)

**********

what does this mean?). If published, this will include your full peer review and any attached files.

If you choose “no”, your identity will remain anonymous but your review may still be made public.

**Do you want your identity to be public for this peer review?**  For information about this choice, including consent withdrawal, please see our Privacy Policy

Reviewer #3: No

**********

[NOTE: If reviewer comments were submitted as an attachment file, they will be attached to this email and accessible via the submission site. Please log into your account, locate the manuscript record, and check for the action link "View Attachments". If this link does not appear, there are no attachment files.]

To ensure your figures meet our technical requirements, please review our figure guidelines: https://journals.plos.org/plosone/s/figures

You may also use PLOS’s free figure tool, NAAS, to help you prepare publication quality figures: https://journals.plos.org/plosone/s/figures#loc-tools-for-figure-preparation.

NAAS will assess whether your figures meet our technical requirements by comparing each figure against our figure specifications.

# Author comment: Author response to Decision Letter 3

**DOI:** 10.1371/journal.pone.0361602.r006

8 Oct 2026

Editor Comments:

Having carefully reviewed the revised manuscript and the authors' point-by-point response, I am satisfied that the majority of the concerns raised in the previous round have been adequately addressed. The taxonomic nomenclature has been corrected throughout the text, tables, and figures in accordance with current bacterial taxonomy; statistical results for non-significant group comparisons have been added; the sequence data accession is appropriately reported in the Data Availability Statement; and a supplementary functional analysis (HUMAnN 3.0) has been incorporated to partially address the concern regarding analytical resolution.

However, while the authors provided a sound methodological justification in their response letter for restricting the association analysis to the genus level and for not incorporating additional public datasets, this justification has not been reflected in the manuscript itself. I therefore ask the authors to add a brief statement to the Limitations section acknowledging the genus-level resolution of the analysis and the potential constraints this, together with the sample size, places on statistical power and the generalizability of the findings. Pending this minor addition, the manuscript will be suitable for acceptance.

Response:

In accordance with the Editor's recommendation, we have added a dedicated statement to the Limitations section of the manuscript to explicitly acknowledge the genus-level resolution and the rationale for not merging our cohort with external public datasets, as well as the resulting constraints on statistical power and generalizability. The integrated paragraph now reads as follows (newly added text is enclosed in square brackets):

Several limitations of this study should be noted. First, our SES indicators did not include dietary exposures and oral health—two closely intertwined factors that shape the microbiome through a bidirectional cycle. Poor oral health can restrict dietary choices, while inadequate nutrition can exacerbate oral diseases; both may substantially affect microbiome composition and developmental processes, thereby influencing a child's cognitive function. Future work should incorporate these variables as key mediators and modifiable risk factors. Second, the cross-sectional design precludes conclusions about causality or developmental trajectories. Longitudinal studies are needed to clarify the directionality and persistence of associations between the microbiome and cognitive performance. [Third, our taxonomic profiling and association analyses were intentionally restricted to the genus level. While this approach was adopted as a conservative strategy to ensure robust classification, mitigate severe zero-inflation, and reduce dimensionality to maintain statistical power, it inherently limits the taxonomic resolution of our findings regarding specific microbial species. Fourth, while a larger sample size obtained by incorporating public datasets would be highly desirable to maximize statistical power, the moderate size of our current cohort was a necessary trade-off. The unique and multi-faceted nature of our dataset—which concurrently captures individual-level data on the oral microbiome, SES, and normative cognitive functioning—made the integration of external data unfeasible without introducing unmanageable confounding factors.] Fifth, the sample was limited to Hispanic and Black American children from the greater NYC area, which may constrain the generalizability of the findings, particularly regarding associations between cognitive performance and specific microbial taxa and their communities, as such relationships may vary across regions. Finally, interpretation is limited by the relatively small body of existing research on the interplay among the oral microbiome, SES, and cognition.

Reviewers' comments: No further comments.

Attachment Submitted filename: Response to Reviewers_PONE-D-25-36917_Revision3.docx

# Editor report: Decision Letter 3

**DOI:** 10.1371/journal.pone.0361602.r007

**Authors:** Antonio Magan-Fernandez

13 Oct 2026

Salivary microbiome, socioeconomic status, and cognitive performance: Triangulating the relationship

PONE-D-25-36917R3

Dear Dr. Naumova,

We’re pleased to inform you that your manuscript has been judged scientifically suitable for publication and will be formally accepted for publication once it meets all outstanding technical requirements.

Within one week, you’ll receive an e-mail detailing the required amendments. When these have been addressed, you’ll receive a formal acceptance letter and your manuscript will be scheduled for publication.

An invoice will be generated when your article is formally accepted. Please note, if your institution has a publishing partnership with PLOS and your article meets the relevant criteria, all or part of your publication costs will be covered. Please make sure your user information is up-to-date by logging into Editorial Manager at Editorial Manager® and clicking the ‘Update My Information' link at the top of the page. For questions related to billing, please contact billing support.

If your institution or institutions have a press office, please notify them about your upcoming paper to help maximize its impact. If they’ll be preparing press materials, please inform our press team as soon as possible -- no later than 48 hours after receiving the formal acceptance. Your manuscript will remain under strict press embargo until 2 pm Eastern Time on the date of publication. For more information, please contact onepress@plos.org.

Kind regards,

Antonio Magan-Fernandez, DDS, MSc, PhD

Academic Editor

PLOS One

Additional Editor Comments (optional):

I have reviewed the revised manuscript and the authors’ response. The only outstanding request from the previous round, acknowledging in the Limitations section the genus-level resolution of the analysis and the constraints this, together with the sample size, places on statistical power and generalizability, has been fully addressed (lines 304–313). The added text is clear, well justified, and consistent across the response letter and both manuscript versions. I therefore consider the manuscript suitable for publication in PLOS ONE in its current form, and thank the authors for their constructive engagement throughout the review process.

Reviewers' comments:

# Editor report: Acceptance letter

**DOI:** 10.1371/journal.pone.0361602.r008

**Authors:** Antonio Magan-Fernandez

PONE-D-25-36917R3

PLOS One

Dear Dr. Naumova,

I'm pleased to inform you that your manuscript has been deemed suitable for publication in PLOS One. Congratulations! Your manuscript is now being handed over to our production team.

At this stage, our production department will prepare your paper for publication. This includes ensuring the following:

* All references, tables, and figures are properly cited

* All relevant supporting information is included in the manuscript submission,

* There are no issues that prevent the paper from being properly typeset

You will receive further instructions from the production team, including instructions on how to review your proof when it is ready. Please keep in mind that we are working through a large volume of accepted articles, so please give us a few days to review your paper and let you know the next and final steps.

Lastly, if your institution or institutions have a press office, please let them know about your upcoming paper now to help maximize its impact. If they'll be preparing press materials, please inform our press team within the next 48 hours. Your manuscript will remain under strict press embargo until 2 pm Eastern Time on the date of publication. For more information, please contact onepress@plos.org.

You will receive an invoice from PLOS for your publication fee after your manuscript has reached the completed accept phase. If you receive an email requesting payment before acceptance or for any other service, this may be a phishing scheme. Learn how to identify phishing emails and protect your accounts at https://explore.plos.org/phishing.

If we can help with anything else, please email us at customercare@plos.org.

Thank you for submitting your work to PLOS One and supporting open access.

Kind regards,

PLOS One Editorial Office Staff

on behalf of

Prof. Antonio Magan-Fernandez

Academic Editor

PLOS One
